# Recent advances on gene-related DNA methylation in cancer diagnosis, prognosis, and treatment: a clinical perspective

**DOI:** 10.1186/s13148-025-01884-2

**Published:** 2025-05-05

**Authors:** Alessandro Lavoro, Daria Ricci, Giuseppe Gattuso, Federica Longo, Graziana Spoto, Anastasia Cristina Venera Vitale, Maria Chiara Giuliana, Luca Falzone, Massimo Libra, Saverio Candido

**Affiliations:** 1 Department of Biomedical and Biotechnological Sciences, University of Catania, 95123 Catania, Italy; 2 Research Center for Prevention, Diagnosis and Treatment of Cancer, University of Catania, 95123 Catania, Italy

**Keywords:** DNA methylation, Differential methylated region, Cancer, Biomarker, Epigenetics, Therapeutic target, Epidrugs, 5-Azacytidine, Decitabine, CRISPR-Cas9

## Abstract

Recent advances in screening programs and the development of innovative therapeutic strategies have significantly improved the clinical outcomes of cancer patients. However, many patients still experience treatment failure, primarily due to inherent or acquired drug resistance mechanisms. This challenge underscores the urgent need for novel therapeutic targets for the effective treatment of malignancies, as well as cancer-specific biomarkers to enhance early diagnosis and guide interventions. Epigenetic mechanisms, including DNA methylation, have recently garnered growing interest as key regulators of gene expression under both physiological and pathological conditions. Although epigenetic dysregulations are reliable tumor hallmarks, DNA methylation is still not routinely integrated into clinical practice, highlighting the need for further research to translate preclinical findings from the bench to the bedside. On these bases, the present review aims to illustrate the state of the art regarding the role of DNA methylation in cancer, describing the technologies currently available for DNA methylation profiling. Furthermore, the latest evidence on the application of DNA methylation hotspots in cancer diagnosis and prognosis, as well as the impact of epidrugs in cancer care, is discussed to provide a comprehensive overview of the potential clinical relevance of DNA methylation in advancing personalized medicine.

## Introduction

Cancer is one of the leading causes of death worldwide in both industrialized and developing countries, whose burden has increased rapidly over the years due to aging, global population growth, and the incidence of cancer-related risk factors [1]. According to GLOBOCAN estimates, over 19 million new cancer cases and almost 10 million cancer-related deaths occurred in 2022, with lung, stomach, colon, and female breast cancers accounting for the highest percentage of both new cases and cancer deaths [2]. Despite recent advances in cancer prevention and treatment, these epidemiological data highlight the need for accurate and robust biomarkers to improve the early diagnosis of cancer and identify novel therapeutic targets for the development of more effective treatment options.

In this field, many studies have demonstrated the pivotal role of genetic alterations in cancer development and progression. Notably, the accumulation of genetic mutations (e.g., point mutations, inversions, translocations, deletions, and polyploidy) drives the malignant transformation of cancer cells and facilitates the acquisition of cancer-specific hallmarks, including cell immortality, apoptosis inhibition, drug resistance, angiogenesis, invasiveness, and the activation of metastatic processes [3–6]. Besides genetic alterations, epigenetic mechanisms have recently emerged as key regulators of gene expression inducing transcription activation or silencing of cancer-related genes and, consequently, the alteration of signaling pathways involved in the regulation of the cell cycle, differentiation, metabolism, cell growth and proliferation, and DNA repair mechanisms [7–11].

Among epigenetic mechanisms, DNA methylation is one of the most well-studied processes. It consists of the enzymatic transfer of a methyl group (-CH_3_) to the carbon-5 position of the cytosine base within the cytosine-guanine (CpG) dinucleotide, leading to the formation of 5-methylcytosine (5mC) [12]. Numerous studies have demonstrated that DNA methylation plays a crucial role in the regulation of several biological processes, including embryonic development, genomic imprinting, and X-chromosome inactivation (XCI). It also contributes to the maintenance of genomic stability by suppressing transposable elements and repetitive DNA sequences [13, 14]. Conversely, aberrant DNA methylation has been implicated in the silencing of tumor suppressor genes and the activation of oncogenes, suggesting its crucial role in cancer initiation and progression [15–17]. Despite its huge potential, DNA methylation has not yet been routinely used for cancer prediction due to several issues related to the lack of standardized criteria for its application in clinical practice [18]. Therefore, further investigations are necessary to provide robust evidence on the validity of DNA methylation as an accurate cancer-related biomarker and effective therapeutic target.

On these bases, the present review aims to report the current knowledge on the involvement of DNA methylation in tumorigenesis and cancer progression, describing the methodologies, novel available technologies, and bioinformatic tools for the analysis of DNA methylation status. Moreover, we summarize the most advanced applications in the clinical setting, providing an overview of DNA methylation hotspots for cancer diagnosis and prognosis, as well as the impact of epidrugs on cancer care and personalized medicine.

### The functional role of DNA methylation in physiological conditions and tumorigenesis

Epigenetics encompasses a broad range of regulatory mechanisms that induce hereditable and stable changes in gene expression without directly altering the DNA sequence composition, including DNA methylation, post-translational histone modifications, and RNA-based mechanisms [19, 20]. Among these regulatory mechanisms, DNA methylation is one of the most well-characterized processes in both physiological and pathological conditions. DNA methylation is essential for mammalian early embryonic development to regulate parental allele-specific expression of imprinted genes, which are organized into clusters of imprinting control regions (ICRs) containing aberrant methylated regions. These regions exhibit distinct methylation patterns that are critical for determining which allele of an imprinted gene is expressed, either in the germline cells for imprint establishment or in somatic cells for imprint maintenance [21]. Additionally, XCI is closely associated with specific DNA methylation patterns. In particular, once an X chromosome is chosen for inactivation, extensive DNA methylation occurs on this chromosome, especially at CpG islands within promoter regions of genes, ensuring long-term gene silencing on the inactive X (Xi) chromosome. Although DNA methylation is not the initial trigger for XCI, it plays a critical role in maintaining the inactivated state; indeed, as the embryo develops and cells divide, DNA methylation ensures the silencing of Xi chromosome across cell generations [22]. Similarly, DNA methylation is essential for preserving genome stability and integrity by inhibiting the activation of transposable elements [23]. Conversely, aberrant DNA methylation represents an epigenetic hallmark strongly associated with tumor initiation and progression, leading to reduced expression of tumor suppressor genes and the activation of oncogenes. Moreover, DNA methylation dysregulation also influences the remodeling of the tumor microenvironment, metabolic adaptation, drug resistance, immune response, and cancer-related inflammation [24–28].

**Fig. 1:**
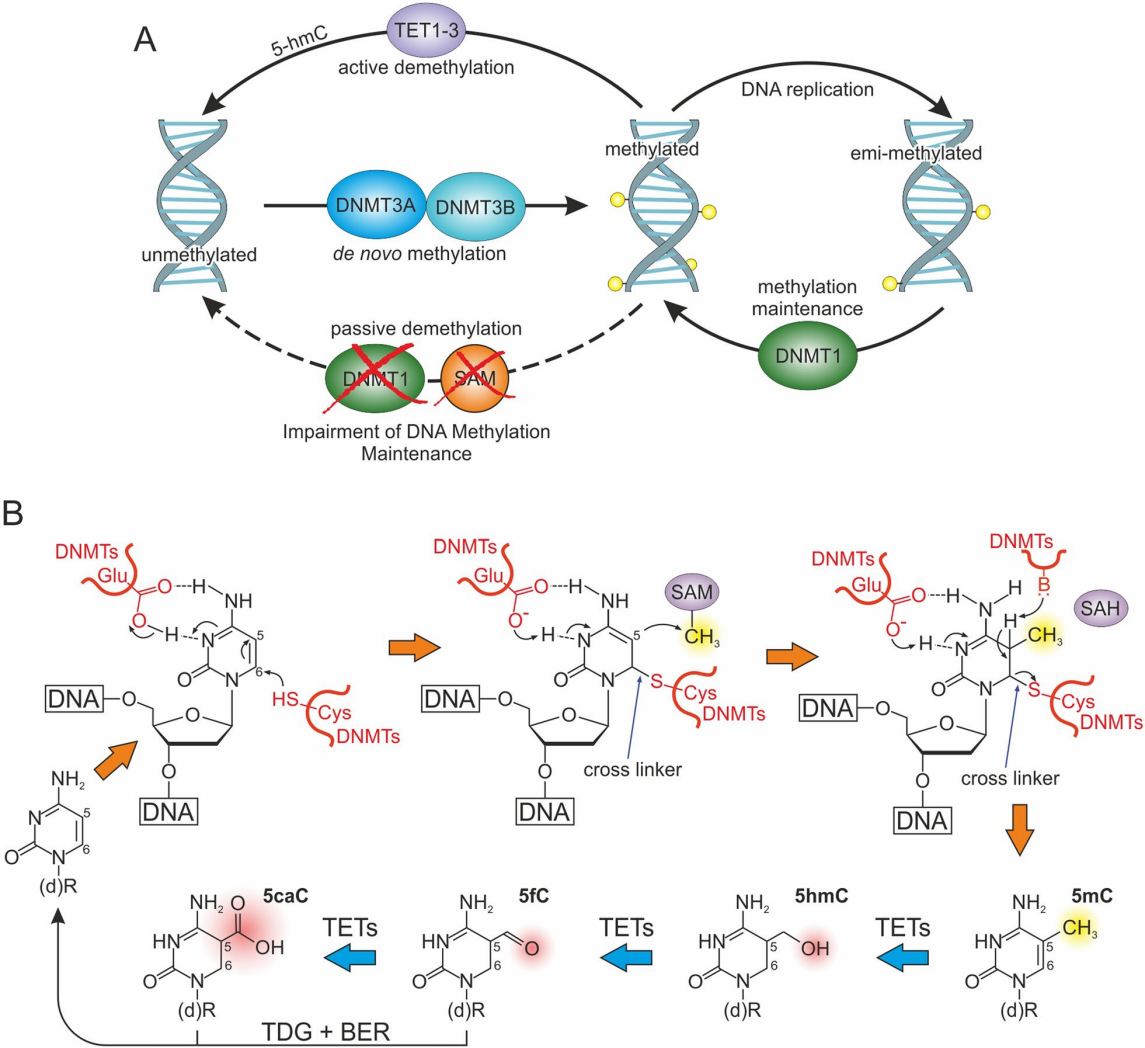
DNA methylation and demethylation dynamics. **A** Schematic representation of DNA methylation/demethylation process. **B** Cycle of DNA methylation and demethylation

As anticipated, DNA methylation involves the transfer of a -CH_3_ group to a DNA sequence using S-adenosyl-L-methionine (SAM) as a donor [29]. The reversible addition of -CH_3_ groups is catalyzed by two enzymatic families, referred to as “writers” and “erasers.” Notably, DNA methyltransferases (DNMTs) are a large group of writer enzymes involved in de novo methylation and its maintenance, whereas Ten-eleven translocation (TET) dioxygenases primarily act as erasers catalyzing DNA demethylation (Fig. 1A) [30].

The DNMT3 family, comprising DNMT3A, DNMT3B, DNMT3C, and DNMT3L, plays a critical role in de novo methylation during embryonic processes. Among these, DNMT3A and DNMT3B, both highly expressed in embryonic stem cells, are the canonical DNMTs catalyzing the establishment of methylation marks on genomic DNA. Conversely, DNMT3L acts as an accessory protein that enhances the methyltransferase activity of DNMT3A/B and has no catalytic function, while DNMT3C has been identified only in rodents as a tandem copy of DNMT3B. The maintenance of methylation status during DNA replication is primarily regulated by DNMT1, which preferentially catalyzes the methylation of hemimethylated DNA (Fig. 1B) [31, 32]. Regarding DNA demethylation, TET family proteins (TET1, TET2, and TET3) are key players of active demethylation, catalyzing the sequential oxidation of 5mC into 5-hydroxymethylcytosine (5hmC), 5-formylcytosine (5fC), and 5-carboxylcytosine (5caC). Passive DNA demethylation may be related to the loss of function of DNMT1 during DNA replication (Fig. 1B) [33, 34].

Along with C5-cytosine methylation, other modifications may occur at N4 of cytosine, N7 of guanine, and N6 of adenine [35]. Among these, DNA methylation at N4-cytosine is found primarily in bacterial and some eukaryotic DNA, whose modifications play a crucial role in pathogen evasion of host immune responses and antibiotic resistance [36]. Conversely, aberrant methylation of N7-guanine is linked to alkylating agent damage, leading to mutations and tumor development [37]. Moreover, N7-guanine modifications have also been involved in the onset of neurodegenerative diseases, including Huntington’s disease [38]. The N6-adenine methylation, mostly studied in prokaryotes, has also been investigated in eukaryotic systems. Specifically, it has been reported that the alteration of DNA methylation status at N6-adenine may have a dual role in cancer, acting either as a tumor promoter or as a suppressor depending on the target gene [39]. Although the studies on these less common DNA modifications have increased over the years, our focus remains on DNA methylation at C5-cytosine, as it is the most relevant epigenetic mechanism involved in tumorigenesis and cancer progression.

**Fig. 2:**
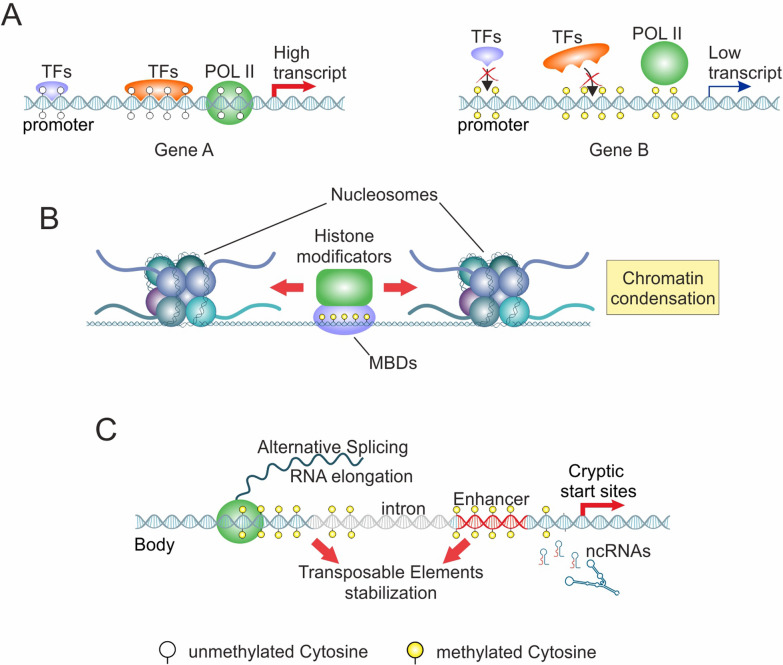
Functional role of DNA methylation in the regulation of gene expression. **A** Schematic representation of promoter hypo- or hypermethylation and transcription activation/inhibition. **B** Recognition of methylated DNA through MBD proteins and chromatin condensation. **C** Schematic representation of intragenic DNA methylation in transcriptional regulation

The CpG dinucleotides are organized into short interspersed DNA sequences (~ 200–1000 bp), known as CpG islands, whose guanine and cytosine (GC) content is greater than 50%. CpG islands are not homogenously distributed across the genome and may be found at significantly higher densities in gene-rich compared to gene-poor areas [40, 41]. CpG islands are typically located within and close to transcription start sites (TSS) of the promoter region (conventionally between 1,000 bp upstream of the TSS and 300 bp downstream of the TSS). CpG islands can be also found in non-promoter regions, such as intragenic CpG islands (from 300 bp downstream of the TSS to 300 bp upstream of the transcription termination site—TTS), 3’-end CpG islands (from 300 bp upstream of the TTS to 300 bp downstream of the TTS), and intergenic CpG islands (from 300 bp downstream of the TTS of one gene to 1,000 bp upstream of the TSS of the following gene) [42, 43]. As widely reported in the literature, aberrant DNA methylation is one of the most significant epigenetic alterations in tumorigenesis, which may affect the expression of tumor suppressor genes and oncogenes. The addition of -CH_3_ groups at the promoter region leads to gene silencing of tumor suppressor genes by inhibiting the binding of transcription factors (TFs) to DNA motifs (Fig. 2A) [44–46].

In this context, methyl CpG-binding domain (MBD) proteins also play a critical role in tumor suppressor silencing by recognizing 5mC and recruiting chromatin modificatory proteins that lead to condensed and closed chromatin, reducing access to TFs and inhibits transcription (Fig. 2B) [47–49]. Conversely, hypomethylation of the promoter region can activate oncogenes, promoting cancer cell proliferation, survival, and metastasis [50–52]. Promoter hypomethylation is also related to the activation of transposable elements, contributing to genomic instability, which in turn promotes tumor initiation and progression. The transposable elements are normally silenced by DNA methylation in heterochromatic regions; however, hypomethylation of these regions can lead to their reactivation. Once activated, transposable elements may insert themselves into regulatory or coding regions, causing insertions and/or deletions that can alter gene function and increase genomic instability. The activation of transposable elements can also induce double-strand breaks, chromosomal rearrangements, translocations, or loss of heterozygosity. Furthermore, the transcription of transposable elements can activate nearby genes or generate novel gene fusions, significantly contributing to tumorigenesis [53, 54].

Although the involvement of promoter DNA methylation in regulating gene expression has been widely demonstrated over the years, the functional significance of intragenic DNA methylation has not yet been completely elucidated. In this field, recent studies have highlighted that intragenic DNA methylation is actively involved in transcriptional regulatory processes [55, 56]. Notably, methylation levels of intragenic CpG islands are higher than those of promoter CpGs during embryonic development, suggesting their potential role in tissue-specific reprogramming [57]. Since open chromatin is typically associated with active transcription and low DNA methylation levels, hypermethylation of the gene body regions in open chromatin sounds as a paradoxical phenomenon. However, actively transcribed genes often exhibit body hypermethylation, which prevents spurious transcription initiation and enhances transcription elongation. Intragenic methylation may also regulate alternative splicing by influencing the binding of splicing factors (e.g., CCCTC-binding factor) and RNA Pol II speed. Furthermore, hypermethylation within body regions has a significant impact on enhancer activity by affecting their accessibility and transcription factor recruitment. Similar to the promoter region, body hypermethylation is a reversible epigenetic hallmark that may serve as a memory mechanism, ensuring hereditable but dynamic gene expression in differentiation and development [58–60]. Interestingly, it has also been reported that body methylation levels are closely associated with DNA replication timing. Previous studies have shown that early- and late-replicating fractions of the genome exhibit distinct methylation profiles. Specifically, early-replicating regions replicate early during S phase of the cell cycle and display higher intragenic methylation levels compared to late-replicating regions. Furthermore, early-replicating regions generally maintain intragenic DNA hypermethylation during cell division, while late-replicating regions undergo gradual demethylation. The positive correlation between DNA replication timing and intragenic methylation suggests that body hypermethylation may contribute to maintaining an open chromatin state, thereby facilitating both replication and transcription [58, 61, 62].

### DNA methylation profiling technologies

Over the years, several technologies have been developed to analyze DNA methylation status. Direct measurement can be achieved using high-performance liquid chromatography (HPLC), high-performance capillary electrophoresis (HPCE), or liquid chromatography coupled with tandem mass spectrometry (LC–MS/MS) [63–65]. Alternatively, indirect approaches for detecting DNA methylation levels include luminometric methylation assay (LUMA) and enzyme-linked immunosorbent assay (ELISA) [66, 67]. However, these approaches only provide a global measurement of DNA methylation and are not suitable for high-throughput processing. Furthermore, these methods are not commonly used due to the large amounts of DNA required for analysis and the need for specialized equipment [68].

The limitations of both direct and indirect methods have been gradually addressed by the introduction of novel approaches for analyzing locus-specific methylation status, including methyl-sensitive PCR and DNA sequencing technologies. Currently, bisulfite conversion is the gold standard pre-processing method for the analysis of methylation status in DNA samples obtained from various biological matrices, including formalin-fixed paraffin-embedded (FFPE) tissues and liquid biopsies. Briefly, this technique converts unmethylated cytosine residues to uracil without affecting the 5mCs. Following bisulfite conversion, methyl-sensitive PCR or DNA sequencing can distinguish between unmethylated cytosines and 5mCs, which appear as thymines and cytosines in the resulting amplified sequence, respectively [69, 70]. Despite its utility for evaluating both whole-genome and locus-specific DNA methylation, bisulfite conversion causes significant fragmentation of the genomic DNA (84–99%), leading to reduced DNA recovery during the cleanup and potentially affecting downstream analyses [71, 72]. An alternative bisulfite-independent method is represented by the methylation-sensitive restriction enzymes (MSRE) assay, which analyzes methylation status using methylation-sensitive restriction enzymes (e.g., *AatII*, *AccII*, *ClaI*, *CpoI*, *HpaII*, and *SmaI*). Notably, these enzymes recognize and cut unmethylated DNA, while the methylated consensus sites are not cleaved. After digestion, DNA is amplified with PCR, and only methylated DNA produces an amplification signal [73, 74]. Although this method does not affect DNA quality, the MSRE assay is not suitable for the analysis of DNA samples with low concentrations due to the large volume of the MSRE digestion mix required for downstream analyses, which can reduce amplification efficiency [75]. In recent years, many studies have proposed novel approaches to address the limitations of standard procedures by combining bisulfite conversion or MSRE digestion pre-processing with droplet digital PCR (ddPCR) amplification [76–82]. Notably, ddPCR has recently emerged as a high-throughput technology for the analysis of low-concentration DNA samples, providing superior sensitivity, linearity, and robustness compared to earlier PCR-based methods [83–88]. Besides ddPCR-based techniques, the advent of novel high-throughput technologies such as sequencing approaches (e.g., next-generation sequencing and pyrosequencing) and microarray (e.g., Infinium HumanMethylation450k Bead Chip array and Infinium Human MethylationEPIC array by Illumina) has revolutionized the analysis of DNA methylation at single-nucleotide resolution [89, 90]. In particular, the EPIC array is an upgraded version of the 450 K BeadChip array and can cover over 850,000 CpG sites, increasing genome coverage of regulatory regions and providing a high-throughput platform for large-scale data generation and comprehensive methylome profiling from diverse biological samples [91]. However, their clinical application remains limited due to the high costs of sequencing procedures and the computational resources required for the identification of DNA methylation hotspots [92].

Other suitable methods for the analysis of DNA methylation, either at a single locus or on a genome-wide scale, include methylcytosine-based DNA immunoprecipitation (MeDIP) and methyl-binding domain capture sequencing (MBDCap-Seq). Notably, MeDIP is based on bridging antibodies that target methylated DNA, whereas MBDCap-Seq relies on MBD proteins [93]. Recently, Nanopore sequencing (Oxford Nanopore Technologies) has emerged as a third-generation technology based on differences in the electric current intensity for the analysis of DNA methylation status. This technology shows many advantages compared to the standard methodologies, including faster analysis, greater data reproducibility, real-time sequencing, lower CG bias, and a higher number of CpG positions called at lower read depth. PCR-free Nanopore sequencing also preserves native DNA modifications, producing reads of thousands of bases in length that enable a more comprehensive methylome analysis. However, the accuracy and precision of Nanopore sequencing in detecting specific methylation sites may vary. Furthermore, interpreting the signal patterns from Nanopore sequencing requires significant computational resources and specialized bioinformatics tools [94, 95].

Despite recent advances in the development of high-throughput technologies for analyzing specific DNA methylation hotspots, further studies are needed to ensure the generation of reproducible and highly sensitive data. Moreover, efforts should focus on establishing standardized protocols for the analysis of biological samples derived from both canonical and non-canonical matrices.

#### Bioinformatic approaches for the analysis of DNA methylation

Although the methodologies described in the previous section enable the detection of differential methylation at single cytosines (DMCs), DNA methylation alterations may also occur across multiple intragenic and intergenic loci, resulting in differentially methylated regions (DMRs). DMRs are genomic regions characterized by distinct DNA methylation patterns between two or more sample groups, such as between different developmental stages, cell types, or diseases versus healthy tissues [96, 97]. Recent advancements in deep sequencing and array-based technologies have enabled the exploration of DNA methylation at a whole-genome or genome-wide scale. Techniques such as whole-genome bisulfite sequencing (WGBS), reduced representation bisulfite sequencing (RRBS), and array-based methods (e.g., Illumina Infinium MethylationEPIC BeadChip) are highly effective for identifying DMRs by comparing DNA methylation patterns across multiple samples. However, the analysis of DNA methylation data remains a challenging task due to intensive bioinformatic resources and expertise in processing big data [98, 99].

In recent years, many computational tools have been developed to facilitate the analysis of DNA methylation data and improve the accuracy of DMRs detection. Among these, Yuditskiy and colleagues introduced BSXplorer, a bioinformatic tool implemented in Phyton for the analysis of bisulfite sequencing data. Specifically, the BSXplorer package is designed to facilitate the graphical exploration of DNA methylation patterns across functional genomic elements and regions of interest to identify DMRs [100]. Similarly, the R-based pipeline PCBS has been proposed as an efficient bioinformatic tool for the analysis of WGBS data and the detection of both differentially methylated loci (DMLs) and DMRs [101]. Another recent open-source tool is DeepMod2, a deep learning framework for DNA methylation analysis using Oxford Nanopore sequencing data. DeepMod2 is optimized to process both POD5 and FAST5 files, providing a fast and accurate model for analyzing various flowcell types, including R10.4 and R9.4 series [102]. Besides the aforementioned pipelines, other bioinformatic approaches have been developed in recent years for the identification of DMRs using array data [103–107]. Among these, methylR is an open-source graphical tool designed for multidimensional analyses of array-based raw data and pathway enrichment analyses [104]. Recently, Yang and Han proposed MethylCallR, a simple and straightforward pipeline for performing epigenome-wide association studies on both public available and custom Methylation BeadChip datasets [107].

Overall, computational approaches enable the processing of large datasets, reducing variability due to human error and enhancing the reliability of findings. Additionally, some tools also support downstream analyses, including gene annotation, correlation between DNA methylation and gene expression, pathway analysis, and visualization, providing deeper insights into biological implications of methylation changes. Despite the great potential of these in silico comprehensive analyses, many pipelines require programming skills that pose a significant barrier for researchers with no bioinformatic expertise. Furthermore, low-quality input data may lead to batch effects, false-positive calls, and inaccurate DMRs identification. In this field, the main challenges are represented by data quality management, standardization of statistical models, and normalization methods across different pipelines and, ultimately, the biological interpretation and validation of findings.

### DNA methylation as a diagnostic and prognostic cancer biomarker

Recent epidemiological data underscore the urgent need for accurate and robust biomarkers to improve the early detection of cancer and enhance the prediction of clinical outcomes. Although hotspot mutation analysis is widely used in clinical and research settings, it is characterized by some limitations that may reduce sensitivity and specificity in cancer diagnosis and prognosis: i) some cancer cells may lack detectable mutations due to intratumor heterogeneity; ii) hotspot mutations are scarce owing to the small quantity of circulating tumor DNA (ctDNA) in comparison with the large amount of normal circulating free DNA (cfDNA) [108]. To address these challenges, many recent studies have focused on DNA methylation, a typical epigenetic hallmark of cancer cells unaffected by tumor heterogeneity, providing a comprehensive view of cancer-related changes across the genome. Notably, it has been reported that the detection of DNA methylation hotspots in both tissue and liquid biopsies may represent a highly sensitive and accurate option not only for early cancer screening but also to provide prognostic information and predict response to therapies [109–111]. Despite recent advancements in the analysis of DNA methylation status and the promising findings of aberrant DNA methylation as a cancer biomarker, its clinical application remains limited due to several issues, including costs compared to current methods, as well as technical challenges related to sensitivity, reproducibility, quantification, and standardization [112]. Therefore, further investigations are needed to achieve a comprehensive validation of methylation-based biomarkers in cancer detection, prognosis, and response to treatment.

In recent years, a growing number of preclinical studies have focused on exploring DNA methylation as a diagnostic and prognostic cancer biomarker. In this regard, we provide a comprehensive overview of the ten most commonly diagnosed cancers worldwide (all cancer cases: 19,964,811) according to GLOBOCAN 2022 report, including lung cancer (LUAD) (12.4%), breast cancer (BRCA) (11.6%), colorectal cancer (CRC) (9.6%), prostate cancer (PRAD) (7.3%), gastric cancer (GC) (4.9%), liver cancer (LIHC) (4.3%), thyroid cancer (THCA) (4.1%), cervical cancer (CC) (3.3%), bladder cancer (BLCA) (3.1%), and non-Hodgkin lymphoma (NHL) (2.8%) [2]. To this purpose, a literature search of the most recent studies (published in 2023 and 2024) was conducted on PubMed public database (https://pubmed.ncbi.nlm.nih.gov/, last accessed October 18, 2024) using the keywords “Methylation” AND “Diagnosis” OR “Prognosis” AND “Cancer type.” Other selection criteria included: (i) the validation study had to be performed using biological samples derived from cancer patients and healthy controls; (ii) the study had to validate specific gene-related DNA methylation hotspots; (iii) the study must report key indicators for assessing the diagnostic accuracy of the investigated biomarkers (sensitivity [SE], specificity [SP], and/or area under the curve [AUC]). Studies with ambiguous data annotations, investigating different tumor types together, published in non‑English language, and duplicates were not included. The adopted approach led to the identification of 138 research articles, of which 81 studies (58.7%) focused on DNA methylation as a diagnostic cancer biomarker, whereas the remaining 57 studies (41.3%) explored the potential of methylation hotspots as cancer-specific prognostic biomarkers. For each tumor type, the main challenges related to the early diagnosis and screening programs are summarized in the following subsections. Furthermore, the most relevant studies for both diagnostic and prognostic applications of DNA methylation are briefly discussed, considering factors as the number of samples and biomarker performance (SE, SP, and AUC) (Table 1).

**Table 1:** Recent studies on DNA methylation as a cancer biomarker. For each study, the relationship between DNA methylation of the investigated genes and diagnostic performance or clinical outcomes are indicated (published during 2023/2024 on https://pubmed.ncbi.nlm.nih.gov/, last accessed October 18, 2024)

Biomarker		Methodology	Sample	Meth. status	Clinical relevance	References
**LUAD: diagnosis**						
*TAC1*, *CDO1*, *HOXA9*, *ZFP42*, *SOX17*, *RASSF1A*,* SHOX2*		qMS-PCR	Blood (*N* = 202)	↑	86.7% SE, 81.4% SP, 0.891 AUC	[113]
*PCDHGA12*,* PRRX1*		Bisulfite-based pyrosequencing, qMS-PCR	Tissue/BW (*N* = 26)	↑	82.4% SE, 87.9% SP, 0.891 AUC	[114]
*PCDHGA12*,* CDO1*		3-plex LTE-qMSP test	BW (*N* = 187)	↑	77.6% SE, 90.3% SP, 0.84 AUC	[115]
*SOX17*, *CDO1*, *TAC1*, *HOXA7*		mdMSP	Plasma (*N* = 72)	↑	90% SE, 82% SP	[116]
*RASSF1A*, *OSR1*, *SHOX2*, *FOXI2*, *NXPH1*, *CDH13*, *SOD3*, *CLEC14A*, *IRX1*, *HOXD9*, *PAX3*, *TBX15*, *DAPK1*,* FOXA1*		multi-locus qPCR assay (LunaCAM)	Plasma (*N* = 172)	↑	50% SE, 94.4% SP, 0.86 AUC	[117]
*HOXA9*		Pyrosequencing	Plasma (*N* = 50)	↑	88% SE, 100% SP, 0.958 AUC	[118]
*RASSF1A*,* ATIC*		MS-HRM	Tissue (*N* = 100)	↑ *RASSF1A* ↓ *ATIC*	*RASSF1A*: 0.71 AUC *ATIC*: 0.80 AUC	[119]
*HOXA7*, *SHOX2*, *SCT*		qMS-PCR	BW (*N* = 329)	↑	74.1% SE, 0.851 AUC	[120]
*HOXB4*, *HOXA7*, *HOXD8*, *ITGA4*, *ZNF808*, *PTGER4*, *B3GNTL1*		AnchorDx EpiVisioTM Indexing PCR Kit	Tissue/plasma (*N* = 317)	↓ *B3GNTL1*, *HOXD8* ↑ *HOXB4*, *ZNF808*, *ITGA4*, *PTGER4*, *HOXA7*	89% SE, 94% SP, 0.97 AUC	[121]
*NCOA2*, *RUNX3*,* BTD*		MS-dPCR	PBMC (*N* = 449)	↑	88.17% SE, 80.2% SP, 0.916 AUC	[122]
**LUAD: prognosis**						
*SNORD3F*		Bisulfite sequencing, real-time PCR	Tissue (*N* = 49)	↑	↓ PFS	[123]
*BMP4*, *HSP90AB1*, *E2F2*,* RXR*		Whole-genome-based methylation assay	CSF (*N* = 10)	↓	↑ OS	[124]
*BCAT1*,* IKZF1*		qMS-PCR	Plasma (*N* = 39)	↑	Disease progression	[125]
*ZNF577*		Pyrosequencing	Tissue (*N* = 100)	↑	↓ OS, DFS	[126]
*SHOX2*, *PTGER4*		SHOX2/PTGER4 DNA Methylation Detection Kit	Blood (*N* = 96)	↑	↑ Treatment Resistance ↓ OS	[127]
*NAA10*		Pyrosequencing	Tissue (*N* = 140)	↓	↓ OS ↓ DFS	[128]
**BRCA: diagnosis**						
*KLRK1*, *KLRD1*, *TRDJ3*, *PLXNA4*		Pyrosequencing, qMS-PCR	PBMC (*N* = 781)	↑	93.2% SE, 90.4% SP, 0.940 AUC	[129]
*MEM132D*,* MYO15B*		MSRE-qPCR	Plasma (*N* = 73)	↓	76% SE, 76% SP, 0.83 AUC	[130]
*CDO1*		MethyLight assay	Tissue (*N* = 281)	↑	88.18% SE, 93.44% SP, 0.96 AUC	[131]
*DOK7*		MSRE-qPCR	Blood (*N* = 60)	↑	89.66% SE, 96.7% SP	[132]
*GCM2*, *ITPRIPL1*, *CACNA1E*, *DLGAP2*		Pyrosequencing, MS-HRM	Tissue (*N* = 144)	↑	97.1% SE, 100% SP, 0.987 AUC	[133]
*RANBP3*, *LCP2*,* GRAP2*		Bisulfite-based PCR	Tissue/Plasma (*N* = 49)	↓	94.1% SE, 93.3 SP, 0.984 AUC	[134]
*CCDC12*		Mass spectometry	Peripheral blood (*N* = 712)	↑	0.727 AUC	[135]
*IL21R*		Mass spectometry	Tissue (*N* = 566)	↓	0.88 AUC	[136]
*SRCIN1*		Infinium MethylationEPIC BeadChip, MS-PCR	Tissue/Plasma (*N* = 245)	↑	78.5% SE, 100% SP, 0.882 AUC	[137]
**BRCA: prognosis**						
*TERT*		Pyrosequencing	Tissue (*N* = 40)	↑	↓ OS	[138]
*ELF5*		qMS-PCR	Plasma (*N* = 100)	↑	↓ OS	[139]
*DACH1*		qMS-PCR	Tissue (*N* = 160)	↑	↓ OS	[140]
*CDH1*		Bisulfite sequencing PCR, MeDIP	Tissue (*N* = 68)	↑	↓ OS	[141]
*BCAT1*, *TBX15*,* CXCL12*		Bisulfite pyrosequencing	Tissue (*N* = 31)	↑ *BCAT1*,* TBX15* ↓ *CXCL12*	↓ OS	[142]
*PTPRO*		MS-PCR	Plasma (*N* = 82)	↓	↑ OS	[143]
*SEPT9*		MS-PCR	Tissue (*N* = 119)	↑	↓ DFS	[144]
**CRC: diagnosis**						
*SDC2*, *SHOX2*		qMS-PCR	Stool/tissue/blood (*N* = 560)	↑	93.83% SE, 92.5% SP, 0.96 AUC	[145]
*SEPT9*, *AXL4*, *SDC2*		qMS-PCR	Plasma (*N* = 940)	↑	82.7% SE, 90.1% SP	[146]
*SDC2*, *ADHFE1*, *PPP2R5C*		qMS-PCR	Stool (*N* = 363)	↑	84.8% SE, 98.0% SP, 0.930 AUC	[147]
*TUSC3*		MethyQESD	PBMC (*N* = 145)	↑	88.6% SE, 76.0% SP, 0.88 AUC	[148]
*AGRN*		MethylTarget sequencing	Plasma (*N* = 118)	↑	75.8% SE, 92.7% SP, 0.85 AUC	[149]
*MLH1*		Amplicon-based NGS	Tissue (*N* = 26)	↑	93% SE, 100% SP	[150]
*SEPT9*, *HIST1H4F*		qDMA-HP	Serum (*N* = 80)	↑	93.3% SE, 81.6% SP, 0.921 AUC	[151]
*FOXF1*		MethyLight PCR	Plasma (*N* = 100)	↑	78% SE, 89.5% SP	[152]
*MMP9*		MethyQESD	PBMC (*N* = 140)	↑	88% SE, 78% SP, 0.976 AUC	[153]
*MGMT*		MethyQESD	PBMC (*N* = 145)	↑	81.43% SE, 75.71% SP, 0.754 AUC	[154]
*SDC2*, *NPY*		multiplex Methyl Light ddPCR	Plasma (*N* = 584)	↑	33–54% SE, 72–96% SP	[155]
*SEPT9*, *BMP3*		ddPCR	Plasma (*N* = 262)	↑	80% SE, 81% SP, 0.845 AUC	[156]
*GALNT9*, *UPF3A*		Methylation EPIC array, Pyrosequencing	Serum (*N* = 433)	↑ *GALNT9* ↓ *UPF3A*	78.8% SE, 100% SP, 0.896 AUC	[157]
*SDC2*,* NDRG4*		MS-PCR	Stool (*N* = 2,333)	↑	92.06% SE, 94.29% SP	[158]
*COL25A1, METAP1D*		MS-ddPCR	Tissue (*N* = 70)	↑	49% SE, 100% SP, 0.79 AUC	[159]
*HAND1*		qMS-PCR	Plasma (*N* = 45)	↑	93.33% SE, 80% SP, 0.8478 AUC	[160]
**CRC: prognosis**						
*CEACAM5*		NGS	Tissue (*N* = 156)	↑	↓ OS	[161]
*BCAT1*, *IKZF1*		qMS-PCR	Plasma (*N* = 142)	↑	↑ Recurrence ↓ OS	[162]
*FLT1*		Infinium Methylation EPIC Array	Tissue (*N* = 110)	↑	↑ Treatment Resistance ↓ OS	[163]
*LINC01594*, *CELF6*, *CD44*		qMS-PCR	Tissue (*N* = 120)	↑ *LINC01594, CELF6* *↓ CD44*	↓ OS	[164]
*APC*		qMS-PCR	Tissue (*N* = 284)	↑	↑ OS	[165]
*MGMT*,* ERCC1*		qMS-PCR	Tissue (*N* = 111)	↑	↑ Chemoresistance ↓ OS	[166]
*MLH1*		Bisulfite sequencing PCR	Tissue (*N* = 580)	↑	↓ OS	[167]
*SCNN1B*		Pyrosequencing	Tissue (*N* = 148)	↑	↓ OS	[168]
**PRAD: diagnosis**						
*RASSF2, GSTP1*, *RASSF1A*		RT-PCR	Plasma/serum (*N* = 54)	↑	*RASSF2*: 69% SE, 39% SP *GSTP1*, *RASSF1A*: 46% SE, 76% SP	[169]
*Cav-1*		Pyrosequencing	Seminal plasma (*N* = 80)	↑	69% SE, 67% SP, 0.63 AUC	[170]
*CCND2*,* GSTP1*		qMS-PCR, NGS	Tissue (*N* = 40)	↑	90% SE, 100% SP, 0.980 AUC	[171]
*LGALS3*		Pyrosequencing	Tissue/blood/seminal plasma (*N* = 97)	↑	56.4% SE, 70.4% SP, 0.664 AUC	[172]
	*PRKY*	qMS-PCR	Tissue (*N* = 33)	↑	96.88% SE, 85.28% SP, 0.952 AUC	[173]
*BRCA1*		qMS-PCR	Tissue (*N* = 40)	↑	0.9043 AUC	[174]
**PRAD: prognosis**						
*PSMA*		WGBS	Tissue (N = 52)	↑	↓ OS	[175]
*WNT5A*		Methylation EPIC array	Tissue (*N* = 8)	↓	↑ Bone metastasis	[176]
*CAMK2N1*		Bisulfite sequencing, pyrosequencing	Tissue (*N* = 68)	↑	↓ OS ↓ PFS	[177]
*APC, RUNX3, GSTP1, TNFRFS10c*,* RASSF1*		Pyrosequencing	Tissue (*N* = 71)	↑	↑ Recurrence	[178]
**GC: diagnosis**						
*RNF180*, *SEPT9*		qMS-PCR	Plasma (*N* = 324)	↑	62.2% SE, 84.8% SP, 0.804 AUC	[179]
*FGFR2*		MSRE-qPCR	Plasma (*N* = 125)	↓	100% SE, 100% SP	[180]
*RUNX3*		MSRE-ddPCR	Serum (*N* = 319)	↑	57.4% SE, 87.6% SP, 0.78 AUC	[181]
*IRF4*,* ZEB2*		qMS-PCR	Plasma (*N* = 114)	↑ *IRF4* ↓ *ZEB2*	74.3% SE, 92.4% SP, 0.85 AUC	[182]
*COL1A1*, *COL1A2*, *COL3A1*,* FN1*		Bisulfite sequencing,	Tissue (*N* = 78)	↓	1.0 AUC	[183]
**GC: prognosis**						
*ZSCAN18*		MassARRAY	Tissue (*N* = 103)	↑	↓ OS	[184]
*PAX5*		PCR	Blood (*N* = 122)	↑	↓ OS	[185]
*MALAT1*,* H19*		Bisulfite-based PCR	Peripheral blood leukocytes (*N* = 150)	↑	↑ Chemotherapy resistance ↓ OS	[186]
*BNIP3*,* DAPK1*		Bisulfite sequencing	Peripheral blood leukocytes (*N* = 250)	↓	↑ Chemotherapy resistance	[187]
*LINE-1*		Bisulfite-based pyrosequencing	Tissue (*N* = 204)	↓	↑ Tumor aggressiveness	[188]
**HCC: diagnosis**						
*RNF135*, *LDHB*		MS-HRM	Plasma (*N* = 726)	↑	0.795 AUC	[189]
*CDKL2*, *USP44*, *ZNF783*		HepaClear	Plasma (*N* = 198)	↑	84.7% SE, 92.0% SP, 0.80 AUC	[190]
*GNB4*, *RNF135*		WGBS, qMS-PCR	Tissue/plasma (*N* = 578)	↑	84.39% SE, 91.92% SP, 0.925 AUC	[191]
*A1AT*, *SERPINA1*		qMS-PCR	Plasma (*N* = 200)	↓	99% SE, 79% SP, 0.93 AUC	[192]
*SEPT9*		SEPT9 assay	Plasma (*N* = 217)	↑	72.82% SE, 89.47% SP, 0.84 AUC	[193]
*GLDC*		qMS-PCR	PBMC (*N* = 197)	↓	72.97% SE, 68.63% SP, 0.782 AUC	[194]
*RASSF1A*, *GSTP1*, *HOXA9*,* ECE1*		qMS-PCR	Urine (*N* = 165)	↑	80% SE, 85% SP, 0.908 AUC	[195]
**HCC: prognosis**						
*SEPT9*		MSRE-ddPCR	Serum (*N* = 157)	↑	↓ OS	[196]
*AHANAK*,* STAP1*		qMS-PCR	PBMC (*N* = 324)	↑	↓ OS	[197]
*ABL1*		qMS-PCR	Tissue (*N* = 99)	↓	↑ Tumor progression	[198]
*NNAT*		BSP	Tissue (*N* = 12)	↑	↓ OS	[199]
*SORT1*		qMS-PCR	Tissue (*N* = 116)	↓	↓ OS	[200]
*APC*		MS-PCR	Serum (*N* = 117)	↑	↑ Tumor progression	[201]
*BTC2*		Pyrosequencing	Tissue (*N* = 51)	↓	↓ OS	[202]
*NLRP7*, *NLRP2*,* NLRP3*		WGBS	Tissue/ buffy coat (*N* = 30)	↓	↓ OS	[203]
**THCA: diagnosis**						
*RUNX1*		MALDI-TOF mass spectrometry	Tissue (*N* = 699)	↓	75% SE, 89% SP, 0.81 AUC	[204]
*MGMT*		ddPCR	Plasma (*N* = 16)	↑	0.8906 AUC	[205]
*TSHR*		qMS-PCR	Plasma (*N* = 103)	↑	86.7% SE, 63.2% SP, 0.719 AUC	[206]
*MAPK14*		MS-HMR	Tissue (*N* = 80)	↓	0.58 AUC	[207]
*FUT4*, *SOCS3*, *IFITM1*, *CD40*, *SLC7A8*		Bisulfite sequencing	PBMC (*N* = 293)	↓	83.33 SE, 90.91% SP, 0.858 AUC	[208]
**THCA: prognosis**						
*ATF3*		qMS-PCR, BSP	Plasma (*N* = 80)	↑	↓ PFS ↓ OS	[209]
*SLC5A8*		qMS-PCR	Tissue (*N* = 165)	↑	↑ Recurrence ↓ OS	[210]
*NDRG4*, *FOXO3*, *ZEB2*, *CDK6*		Infinium HD Methylation 850 K methylation chip, qMS-PCR	Tissue (*N* = 12)	↑ *NDRG4* ↓ *FOXO3*, *ZEB2*, *CDK6*	↓ OS	[211]
*NNAT*		Human Infinium MethylationEPIC BeadChip (Illumina)	Tissue (*N* = 78)	↑	↓ OS	[212]
**CC: diagnosis**						
*ASTN1*, *DLX1*, *ITGA4*, *RXFP3*, *SOX17*,* ZNF671*		qMS-PCR	Tissue (*N* = 61)	↑	85% SE, 85.4% SP, AUC 0.850	[213]
*PAX1*, *ZNF671*		Pyrosequencing, qMS-PCR	Tissue (*N* = 633)	↑	*ZNF671*: 79.46% SE, 79.88%, SP *PAX1:* 74.11% SE, 66.86% SP	[214]
*SEPT9*		qMS-PCR	Tissue/scraping (*N* = 113)	↑	95.52% SE, 68.51% SP, 0.92 AUC	[215]
*PAX1*, *ZNF582*		qMS-PCR	CCB (*N* = 115)	↑	88.24% SE, 91.89% SP	[216]
*PCDHGB7*		MSRE-qPCR	CCB (*N* = 1,135)	↑	82.4% SE, 92.1% SP	[217]
*ADCYAP1*, *BHLHE22*, *CDH13*, *CDO1*, *GALR1*, *GHSR*, *HAND2*, *SST*,* ZIC1*		qMS-PCR	Urine/tissue/scraping (*N* = 420)	↑	90% SE, 90% SP, 0.83 AUC	[218]
*DPP6*, *RALYL*,* GSX1*		Bisulfite-based qPCR	Scraping (*N* = 28,017)	↑	77% SE, 76.9% SP	[219]
*FAM19A4*		qMS-PCR	ThinPrep (*N* = 330)	↑	75% SE, 91% SP	[220]
*CA10*, *DPP10*, *FMN2*,* HAS1*		NGS	ThinPrep (*N* = 373)	↑	84.3% SE, 95% SP, 0.9337 AUC	[221]
*RASSF1A*,* HIST1H4F*		qMS-PCR	Tissue/Pap brush (*N* = 126)	↑	0.945 AUC	[222]
*ASCL1*,* LHX8*		qMS-PCR	LBC (*N* = 411)	↑	84.6% SE, 86.7% SP	[223]
**CC: prognosis**						
*PAX1*		qMS-PCR	Exfoliated cells (*N* = 125)	↓	↑ Radio-resistance	[224]
*NOVA1*, *GSTM5*, *TRHDE*,* CXCL12*		MassARRAY EpiTYPER Assay	Tissue (*N* = 30)	↑	↓ OS	[225]
*EPB41L3*		Pyrosequencing	LBC (N = 343)	↑	↑ Disease severity	[226]
*MGMT*		MS-PCR	Blood (*N* = 48)	↓	↑ OS	[227]
*ASCL1*, *LHX*,* FAM19A4*		qMS-PCR	Scraping (*N* = 364)	↑	↑ Recurrence	[228]
*GHSR*, *SST*,* ZIC1*		qMS-PCR	Urine (*N* = 47)	↑	↑ Recurrence	[229]
**BLCA: diagnosis**						
*ZNF671*, *OTX1*, *IRF8*		qMS-PCR	Urine (*N* = 90)	↑	75% SE, 90.9% SP, 0.91 AUC	[230]
*ONECUT2*		qMS-PCR	Urine (*N* = 203)	↑	100% SE, 68.2% SP	[231]
*HIST1H4F*		Pyrosequencing	Tissue (*N* = 47)	↑	88.64% SE, 88.9% SP, 0.899 AUC	[232]
*PENK*		qMS-PCR	Urine (*N* = 318)	↑	86.9% SE, 91.6% SP, 0.892 AUC	[233]
*TWIST1, VIM*		MethyLight assay	Urine (*N* = 231)	↑	78% SE, 83% SP	[234]
*GHSR*,* MAL*		qMS-PCR	Urine (*N* = 134)	↑	79% SE, 80% SP, 0.87 AUC	[235]
*DAPK*		qMS-PCR	Urine (*N* = 1,021)	↑	92.86% SE, 91.63% SP, 0.922 AUC	[236]
*GATA4*, *P16*, *P14*, *APC*, *CDH1*,* CD99*		MS-PCR	Urine (*N* = 246)	↑	62.9 SE, 98% SP, 0.805 AUC	[237]
*SLC31A1*		Bisulfite sequencing	Tissue (*N* = 50)	↓	0.90 AUC	[238]
*NRN1*		Bisulfite sequencing, MS-PCR	Tissue (N = 240)	↑	85% SE, 86% SP, 0.9391 AUC	[239]
**BLCA: prognosis**						
*MIR145*		Bisulfite-based pyrosequencing	Plasma (*N* = 140)	↓	↓ DFP	[240]
*MAPK14*		Infinium Methylation EPIC Bead Chips (Illumina, Inc.)	Blood (*N* = 601)	↓	↓ OS	[241]
*FGFR3*,* TP53*		MS-PCR	Tissue (*N* = 115)	↑ *FGFR3* ↓ *TP53*	↓ RFS ↓ PFS	[242]
*PENK*		me*PENK* test	Urine (N = 186)	↑	↓ RFS ↓ OS	[243]
*VIM*		qMS-PCR	Tissue (*N* = 144)	↓	↑ Tumor aggressiveness	[244]
*TERT*		MS-PCR	Tissue (*N* = 100)	↑	↓ OS	[245]
**NHL: diagnosis**						
*HLX-AS1*, *CHST15*, *MIR12123*, *DLK1*, *LINC02115*, *MIR3973*, *NCAM2*		Full epigenetic (EPIC) methylation sequencing	Plasma (*N* = 1,328)	↑	95.3% SE, 95.8% SP, 0.994 AUC	[246]
*BILF2*		qMS-PCR	NB (*N* = 136)	↓	98.81% SE, 76.92% SP, 0.9801 AUC	[247]
**NHL: prognosis**						
*NDRG2*		qMS-PCR	Tissue (*N* = 64)	↓	↑ OS	[248]
*BCL11A, IRF4*,* BCL6*		Bisulfite-based Pyrosequencing	Tissue (*N* = 64)	↑	↓ OS	[249]
*TRIP13*		WGBS	Tissue (N = 9)	↓	↓ OS	[250]

(↑: Increased; ↓: Decreased)

#### Lung cancer

The diagnosis of LUAD is currently based on low-dose computed tomography (LDCT) screening. Although LDCT is considered the gold standard method, it has significant drawbacks and challenges, including false positives, overdiagnosis, and associated costs [251]. Consequently, there is an urgent need for more robust and effective non-invasive diagnostic tools to improve the early detection of LUAD by identifying valuable cancer-specific biomarkers in blood samples or bronchial lavage.

In this context, several studies have investigated DNA methylation as a diagnostic and prognostic biomarker for LUAD. As summarized in Table 1, 16 preclinical studies met the aforementioned criteria, of which 10 examined diagnostic accuracy and six focused on prognostic value. Notably, the aberrant methylation of *SHOX homeobox 2* (*SHOX2*), *cysteine dioxygenase 1* (*CDO1*), *Ras association domain family member 1A* (*RASSF1A*), and *homeobox A7* (*HOXA7*) genes was explored in at least three different studies. Among the most representative preclinical studies, Du and colleagues recently proposed methylation analysis of a panel of genes, including *RASSF1*A, *CDO1, SRY-box transcription factor 17* (*SOX17*)*, homeobox A9* (*HOXA9*)*, tachykinin precursor 1* (*TAC1*)*, SHOX2,* and *ZFP42 zinc finger protein* (*ZFP42*), for the early detection of LUAD [113]. Notably, increased methylation levels of these genes enabled the differentiation of LUAD patients from benign nodules and healthy individuals, achieving high SE (86.7%), SP (81.4%), and AUC (0.891) in blood samples [113]. Similarly, Hu et al. performed targeted bisulfite sequencing on tissue and plasma samples (*N* = 317) from LUAD and benign lung disease patients, identifying seven differentially methylated genes by analyzing 9,307 DMRs [121]. Specifically, hypomethylation of *UDP-glucose ceramide glucosyltransferase like 1* (*B3GNTL1*) and *homeobox D8* (*HOXD8*), combined with hypermethylation of the *homeobox B4* (*HOXB4*), *HOXA7*, *integrin subunit alpha 4* (*ITGA4*), *zinc finger protein 808* (*ZNF808*), and *prostaglandin E receptor 4* (*PTGER4*) genes, demonstrated significant accuracy for the detection of early-stage LUAD patients [121]. Another recent study validated a diagnostic panel of genes with aberrant DNA methylation. Using peripheral blood mononuclear cells (PBMCs) samples, methylation-specific chip-based digital PCR (MS-dPCR) analysis revealed that hypermethylation of *nuclear receptor coactivator 2* (*NCOA2*), *RUNX family transcription factor 3* (*RUNX3*), and *biotinidase* (*BTD*) exhibited high diagnostic performance in detecting stage I LUAD patients (SE: 88.17%; SP: 80.2%; AUC: 0.916) [122].

Although aberrant DNA methylation appears to be closely associated with the early detection of LUAD, several studies also explored the potential application of DNA methylation hotspots as prognostic biomarkers. For instance, the aberrant methylation of the *zinc finger protein 577* (*ZNF577*) gene has been proposed as a promising epigenetic biomarker for predicting clinical outcomes in LUAD patients. Specifically, Munkhjargal and colleagues performed pyrosequencing analysis of tissue biopsy samples (*N* = 100) and demonstrated that *ZNF577* promoter hypermethylation was strongly associated with reduced mRNA levels and unfavorable prognosis in LUAD patients [126]. According to the aforementioned studies, hypermethylation of *SHOX2* and *PTGER4* has been suggested as a valuable diagnostic biomarker for LUAD. However, both genes have also been proposed as potential prognostic biomarkers to differentiate between responders and non-responders to first-line treatment, including chemotherapy + / − radiotherapy, anti-*epidermal growth factor receptor* (*EGFR*) therapy, and immunotherapy [127]. Notably, Fleischhacker et al. highlighted that increased methylation levels of *SHOX2* and *PTGER4* at the time of re-staging were significantly related to poor clinical outcomes in LUAD patients [127]. The aberrant DNA methylation of *N-Alpha-Acetyltransferase 10* (*NAA10*), a key regulator of cell growth and apoptosis, has recently emerged as a LUAD-specific prognostic biomarker. Specifically, it has been reported that *NAA10* promoter methylation levels were significantly lower in LUAD patients compared to healthy controls (17.5% *vs* 66.8%, respectively) and inversely correlated with mRNA expression [128]. Furthermore, Lee and colleagues demonstrated that LUAD patients with *NAA10* hypomethylation had decreased survival rates compared to those with *NAA10* hypermethylation, underscoring its clinical value as a prognostic biomarker [128].

#### Breast cancer

BRCA is predominantly characterized by asymptomatic progression, underscoring the need for highly sensitive and specific biomarkers to enable early detection and effective management of cancer patients using minimally invasive methods [252]. In the last year, the clinical utility of DNA methylation has been extensively explored, with nine validation studies performed on tissue and plasma samples from BRCA patients and healthy controls. Similarly, seven preclinical prognostic investigations have been conducted, primarily focusing on the methylation status of specific genes. Notably, only one prognostic study examined a panel of three genes, including *branched-chain amino acid transaminase 1* (*BCAT1*), *T-box transcription factor 15* (*TBX15*), and *C-X-C motif chemokine ligand 12* (*CXCL12*) [142], while an equal number of diagnostic studies assessed the DNA methylation status of gene-related panels and single genes (Table 1).

Among the studies with the highest diagnostic performance and number of analyzed samples, Wang and colleagues identified and validated four DNA methylation hotspots belonged to immune receptor-associated genes: *killer cell lectin-like receptor K1* (*KLRK1*), *killer cell lectin-like receptor D1* (*KLRD1*), *T-cell receptor delta joining 3* (*TRDJ3*), and *plexin A4* (*PLXNA4*) [129]. The identified methylation hotspots, all mapped into the TSS1500 region of the respective genes, were hypermethylated in BRCA patients compared to healthy controls, demonstrating high diagnostic accuracy for detecting early-stage and minimal BRCA tumors (SE: 93.2%; SP: 90.4%; AUC: 0.94) [129]. Another recent study also proposed the methylation analysis of the *CDO1* gene as a promising epigenetic biomarker for the early detection of BRCA [131]. Specifically, Yang and colleagues found that the DNA methylation levels of the *CDO1* promoter were significantly increased in BRCA tissue samples compared to control group (mean methylation levels: 7.46% ± 15.38 *vs* 0.19% ± 0.83, respectively) [131]. Krasnyi and colleagues also evaluated the methylation status of a panel of four genes, including *glial cells missing homolog 2* (*GCM2*)*, inositol 1,4,5-trisphosphate receptor-interacting protein like 1* (*ITPRIPL1*)*, calcium voltage-gated channel subunit alpha1 E* (*CACNA1E*), and *discs large homolog-associated protein 2* (*DLGAP2*), in a cohort of BRCA patients (*N* = 96) and controls (*N* = 24 fibroadenoma tissue samples and 24 healthy tissue samples) [133]. Interestingly, pyrosequencing analysis combined with methylation-sensitive high-resolution melting (MS-HRM) assay revealed that the simultaneous hypermethylation of these four genes could serve as a valuable epigenetic biomarker for distinguishing BRCA patients from fibroadenoma patients and healthy individuals (SE: 97.1%; SP: 100%; AUC: 0.987) [133].

The relationship between DNA methylation profiling and the clinical outcomes of BRCA patients has been extensively investigated in recent years. Interestingly, promoter hypermethylation of *E74-like ETS transcription factor 5* (*ELF5*) was recently identified as a valuable epigenetic biomarker for predicting clinical outcomes in BRCA patients [139]. Specifically, Salimi et al. observed that the *ELF5* promoter was completely unmethylated in the control group, whereas its hypermethylation was strongly associated with regional lymph node involvement and distant metastasis, ultimately correlating with poorer prognosis of BRCA patients [139]. A recent study also demonstrated that promoter hypermethylation of *Dachshund family transcription factor 1* (*DACH1*) was related to its silencing and worsened prognosis for BRCA patients [140]. The authors demonstrated that the increased methylation levels of the *DACH1* promoter region were associated with triple-negative and stage IV BRCA with distant metastasis, while no methylation was detected in normal adjacent tissues [140]. Furthermore, the *Septin 9* (*SEPT9*) gene, whose aberrant methylation has been widely investigated in various tumor types, was recently validated as a robust prognostic predictor for BRCA patients [144]. In this regard, Zhang and colleagues reported that BRCA patients with *SEPT9* hypermethylation were characterized by reduced disease-free survival (DFS) rates compared to those with no *SEPT9* methylation (mean DFS: 20.2 months *vs* 53.5 months, respectively) [144].

#### Colorectal cancer

Currently, colonoscopy is the gold standard for CRC screening and diagnosis. Despite the effectiveness of this invasive procedure, there is an urgent need for novel and non-invasive diagnostic methods to improve adherence to screening programs and facilitate the identification of high-risk individuals, who can then be referred for more in-depth CRC diagnostic examinations [253]. To this purpose, an increasing number of studies have focused on DNA methylation as a non-invasive and highly promising approach. As reported in Table 1, among the analyzed tumor types, CRC shows the highest number of preclinical studies on DNA methylation (*N* = 24), including 16 diagnostic and eight prognostic studies. Interestingly, *Syndecan 2* (*SDC2*) and *SEPT9* were the most frequently explored genes, featured in five and three distinct studies, respectively.

Among the most relevant studies conducted in the last year, Shen and colleagues recently validated *SDC2* and *SHOX2* DNA methylation hotspots across different biological matrices (tissue, blood, and stool samples) as diagnostic biomarkers for the early detection of CRC [145]. Notably, cg13096260, located in the promoter region of the *SDC2* gene, and cg12993163, mapped into the body region of *SHOX2*, were significantly hypermethylated in CRC stool samples, demonstrating high diagnostic performance (SE: 93.83%; SP: 92.5%; AUC: 0.96) [145]. Similarly, Li et al. investigated the diagnostic potential of *SDC2* in combination with other cancer-related genes, including *alcohol dehydrogenase iron containing 1* (*ADHFE1*) and *protein phosphatase 2 regulatory subunit B'gamma* (*PPP2R5C*) [147]. Using the quantitative methylation-specific PCR (qMS-PCR) analysis, the authors revealed that DNA methylation levels of this gene-based panel were significantly increased in stool samples from CRC patients compared to those with advanced adenoma, non-advanced adenoma, hyperplastic or other polyps, and no-disease patients (SE: 84.8%; SP: 98%; AUC: 0.930) [147]. The methylation analysis of *polypeptide N-acetylgalactosaminyltransferase 9* (*GALNT9*) and *UPF3 regulator of nonsense-mediated MRNA decay homolog A* (*UPF3A*) has been also reported as a highly sensitive and specific blood-based diagnostic test for CRC screening [157]. Specifically, Gallardo- Gómez et al. demonstrated that *GALNT9* (cg11113216) hypermethylation, combined with *UPF3A* (cg01550272) hypomethylation, enabled discrimination of advanced CRC from benign pathologies with 78.8% SE and 100% SP (AUC: 0.896) [157].

Besides the diagnostic potential, the prognostic significance of DNA methylation has also been explored for CRC. Among the most notable studies, Huang and colleagues recently proposed *CEA Cell Adhesion Molecule 5* (*CEACAM5*) as a promising prognostic tumor marker for CRC, whose aberrant DNA methylation showed a strong correlation with disease prognosis [161]. Specifically, the authors reported that hypermethylation of CpG regions within the *CEACAM5* promoter was closely related to *B-Raf proto-oncogene* (*BRAF*) mutation, *transforming growth factor beta 2* (*TGFB2*) mutation, and microsatellite instability, leading to worst OS in CRC patients [161]. Recently, Ali et al. also investigated the prognostic value of *speckle-type Poz protein* (*SPOP*) and *Adenomatous Polyposis Coli* (*APC*) gene alterations in an Indian population of CRC patients [165]. Interestingly, CRC patients with moderately or poorly differentiated tumors, as well as those with lymph node metastases, have increased methylation levels in the *APC* promoter. Moreover, *APC* hypermethylation and *SPOP* downregulation were significantly correlated with improved survival rates [165]. Furthermore, Tan and colleagues identified five regions mapped into *MutL homolog 1* (*MLH1*) promoter (A, B, C, D, and E) as prognostic biomarkers for Chinese CRC patients [167]. Notably, these regions were predominantly methylated in elderly (aged > 50 years) female CRC patients with no family history of related tumors, which showed reduced overall survival (OS) rates compared to unmethylated CRC patients [167].

#### Prostate cancer

The prostate-specific antigen (PSA) test in blood samples is routinely used in screening programs for the early detection of PRAD. However, high PSA levels may be also observed in other pathological conditions, including benign prostate hyperplasia (BPH), prostatitis, and urinary tract infections, leading to false positive [254]. Consequently, recent studies have focused on the identification of alternative PRAD biomarkers to overcome the limitations of PSA test.

As reported in Table 1, a reduced number of recent preclinical studies have been conducted to assess the diagnostic and prognostic values of DNA methylation in PRAD (*N* = 10). Among these, Zhang and colleagues validated an in silico identified dual-gene signature for DNA methylation analysis in PRAD and adjacent normal tissues [171]. Notably, the authors reported that promoter hypermethylation of *cyclin-D2* (*CCND2*) and *glutathione S-transferase pi-1* (*GSTP1*) enabled the screening of PRAD patients with high accuracy (SE: 90%; SP: 100%; AUC: 0.98). Moreover, the hypermethylation of both genes was significantly correlated with reduced immune cell infiltration and disease progression, suggesting their clinical value for the management of PRAD patients [171]. Another recent study investigated the diagnostic potential of *lectin galactoside-binding soluble 3* (*LGALS3*) methylation analysis, also known as *galectin 3* [172]. Interestingly, the methylation analysis of tissue and seminal plasma samples showed a significant difference between PRAD and BPH groups. In particular, promoter CpG islands of *LGALS3* had increased methylation levels in PRAD compared to BPH patients, highlighting that *LGALS3* methylation analysis could serve as a highly effective liquid biopsy-based diagnostic test outperforming PSA [172]. Recently, the aberrant methylation of the *Protein Kinase Y-Linked* (*PRKY*) gene, located on the Y-chromosome, has emerged as a promising and valuable diagnostic marker for the early detection of PRAD [173]. Specifically, Dai et al. demonstrated that the hypermethylation of the *PRKY* promoter (cg05163709, cg08045599, and cg05618150) showed high diagnostic accuracy in detecting PRAD at early stages (SE: 96.88%; SP: 85.28%; AUC: 0.952) [173].

Regarding DNA methylation biomarkers for PRAD prognosis, only four studies met the selection criteria described above. Among these, Sayar et al. explored the relationship between *prostate-specific membrane antigen* (*PSMA*) methylation and relative gene expression in metastatic castration-resistant prostate cancer (mCRPC) [175]. The authors demonstrated that *PSMA* downregulation is due to the gain of CpG methylation, which was also associated with disease progression and reduced OS in mCRPC patients [175]. Interestingly, Peng et al. explored the epigenetic mechanisms involved in the regulation of *calcium-/calmodulin-dependent protein kinase II inhibitor I* (*CAMK2N1*), a tumor suppressor gene significantly downregulated in PRAD [177]. Specifically, the authors performed in vitro functional studies by treating DU145 and PC-3 cells with the demethylating agent 5-azacytidine-2′-deoxycytidine (5-Aza-CdR) and demonstrated that *CAMK2N1* promoter demethylation was related to mRNA overexpression. Furthermore, the methylation analysis of PRAD tissues revealed that *CAMK2N1* promoter hypermethylation (from cg14477205 to cg24294857) was closely linked to advanced disease (Stages III/IV) and reduced progression-free survival (PFS) [177]. Recently, Eismann and collaborators analyzed the methylation status of various gene loci, including *GSTP1*, *Ras association domain family member 1* (*RASSF1)*, *TNF receptor superfamily member 10b* (*TNFRFS10c*), *RUNX3*, and *APC*, in cancerous and adjacent benign tissues [178]. In particular, the DNA methylation status of the analyzed loci showed significant differences between high-risk and low-risk patients, with higher methylation levels observed in high-risk patients. Furthermore, the hypermethylation of *APC* in PRAD tissues was closely associated with an increased risk of biochemical recurrence (BCR) after radical prostatectomy (RP) [178].

#### Gastric cancer

The esophagogastroduodenoscopy (EGD) remains the primary diagnostic procedure for GC. Although EGD is considered a highly accurate method for directly visualizing the stomach lining and obtaining biopsies of suspicious lesions, it is generally invasive and uncomfortable for patients [255]. Therefore, the development of non-invasive or minimally invasive diagnostic procedures, along with the identification of novel biomarkers, is crucial to minimize discomfort and significantly improve the early diagnosis of GC.

Recent studies in this field have evaluated the efficacy of DNA methylation hotspots as non-invasive diagnostic biomarkers in liquid biopsy samples. However, only five diagnostic studies met the adopted criteria (Table 1). Among these, Nie and colleagues focused on aberrant methylation of *ring finger protein 180* (*RNF180*) and *SEPT9*, assessing their potential as cancer-specific biomarkers both individually and in combination [179]. The qMS-PCR analysis of plasma samples revealed that the simultaneous hypermethylation of the two genes yielded the most promising results (SE: 62.2%; SP: 84.8%; AUC: 0.804) compared to *RNF180* alone (SE: 46.2%; SP: 87.3%; AUC: 0.723) and *SEPT9* alone (SE: 40%; SP: 96.0%; AUC: 0.741) [179]. Similarly, DNA methylation status of *RUNX3*, a tumor suppressor gene significantly downregulated in GC, was also investigated in tumor context by Nakamura et al. to identify a novel epigenetic biomarker [181]. Notably, the authors reported that *RUNX3* methylation levels were significantly higher in serum samples from GC patients (*N* = 94) compared to healthy controls (*N* = 225), providing a valuable alternative to EGD for GC diagnosis (SE: 57.4%; SP: 87.6%; AUC: 0.78) [181]. Another recent study proposed a dual-gene methylation signature, including *interferon regulatory factor 4* (*IRF4*) and *zinc finger E-box binding homeobox 2* (*ZEB2*), for potential clinical application in the early detection of GC [182]. Interestingly, Bu and colleagues identified a marker combination comprising two DNA methylation hotspots belonging to *IRF4* (cg06223767 and cg05766140) and one from *ZEB2* (cg16405026), whose aberrant methylation in plasma samples showed high diagnostic performance (AUC: 0.85) for GC diagnosis [182].

Besides its diagnostic potential, aberrant DNA methylation of specific CpGs has been also proposed as a predictive and prognostic GC biomarker. For instance, Wang and colleagues recently investigated the significance of DNA methylation alterations in the *metastasis-associated lung adenocarcinoma transcript 1* (*MALAT1*) and *H19 imprinted maternally expressed transcript* (*H19*) long non-coding RNA for predicting chemotherapy efficacy in GC patients [186]. Notably, methylation analysis revealed that hypermethylation of cg06197492, localized within the *H19* body region, combined with hypermethylated cg12498916, mapped into the *MALAT1* TSS200 region, was closely related to chemotherapy resistance and poorer OS in GC patients [186]. A recent case–control perspective study, involving 150 GC patients and 100 healthy subjects, explored the prognostic value of a two-gene panel, including *BCL2 interacting protein 3* (*BNIP3*) and *death-associated protein kinase 1* (*DAPK1*) [187]. The authors demonstrated that promoter hypomethylation of both genes was inversely correlated with mRNA expression and chemotherapy efficacy. Conversely, a positive correlation was observed between *BNIP3* and *DAPK1* methylation status and immune cell infiltration, leading to poorer OS in GC patients [187]. Furthermore, Baba and colleagues evaluated the role of DNA methylation in the regulation of *long interspersed nuclear element-1* (*LINE-1*) to identify novel DNA methylation biomarkers related to GC development and progression [188]. Notably, bisulfite-based PCR analysis highlighted that *LINE-1* hypomethylation was associated with aggressive and advanced GC, suggesting its clinical value as a predictive GC-specific epigenetic biomarker [188].

#### Liver cancer

Among LIHCs, hepatocellular carcinoma (HCC) is the most common type, accounting for 75% of all cases. The intricate etiology and heterogeneity of HCC pose significant challenges to its early diagnosis, and most patients are diagnosed at advanced stages [256]. Currently, the primary strategy for HCC surveillance involves a combination of imaging techniques (e.g., ultrasound) and blood tests (e.g., alpha-fetoprotein—AFP test). However, both ultrasound and the AFP test exhibit low SE (47% and 40%, respectively) for detecting early-stage HCC [257].

In recent years, several preclinical studies have investigated the potential use of DNA methylation hotspots as diagnostic and prognostic biomarkers to improve the management of HCC patients. Based on the selection criteria, a total of 15 preclinical studies are summarized in Table 1, including seven diagnostic and eight prognostic studies. Among the most relevant studies, Bay and colleagues developed a novel blood-based diagnostic panel (HepaClear), which combined methylation analysis of three gene-related CpG sites with AFP and des-gamma-carboxyprothrombin (DCP) protein markers [190]. Specifically, the hypermethylation of *cyclin-dependent kinase like 2* (*CDKL2*—cg14263942), *ubiquitin-specific peptidase 44* (*USP44*—cg12701184), and *zinc finger protein 783* (*ZNF783*—cg14570307) enabled the detection of early-stage HCC, achieving 84.7% SE and 92% SP [190]. Similarly, Zhao and colleagues proposed a dual-marker panel based on methylation analysis of *guanine nucleotide-binding protein subunit beta-4* (*GNB4*) and *ring finger protein 135* (*RNF135*) in tissue and plasma samples for the early detection of HCC [191]. Notably, the authors demonstrated that *GNB4* and *RNF135* hypermethylation exhibited higher diagnostic accuracy for detecting HCC compared to the standard AFP test (SE: 84.39%; SP; 91.92%; AUC: 0.925) [191]. In this context, another recent study validated a panel of four genes with DMRs, including *RASSF1A*, *GSTP1*, *HOXA9*, and *endothelin-converting enzyme 1* (*ECE1*), as a non-invasive diagnostic tool for HCC [195]. In particular, Lin and colleagues demonstrated that increased methylation levels of these four markers, when combined with the AFP test, had higher diagnostic accuracy in detecting HCC compared to AFP alone (SE: 80% *vs* 29.5%; SP: 85% *vs* 95.4%; AUC: 0.908 *vs* 0.841) [195].

Despite many recent studies have focused on analyzing specific DNA methylation patterns as potential prognostic biomarkers for HCC, most investigations lack strong evidence based on clinical samples. Indeed, only eight preclinical studies met the selection criteria (Table 1). Among the selected studies, Saeki and colleagues evaluated the relationship between *SEPT9* methylation and clinical outcomes in HCC patients [196]. Notably, *SEPT9* hypermethylation, combined with high plasma levels of AFP (≥ 400 ng/mL), was identified as a predictor of poor OS in HCC patients receiving sorafenib or lenvatinib as first-line therapy [196]. Another recent study focused on the aberrant DNA methylation of *AHNAK nucleoprotein* (*AHANAK*) and *signal transducing adaptor family member 1* (*STAP1*) in the diagnosis and prognosis of HBV-related hepatopathy [197]. Specifically, Li and colleague conducted an observational study involving 324 subjects (46 chronic hepatitis B, 46 compensatory liver cirrhosis, 53 decompensated liver cirrhosis, 157 HCC, and 22 healthy controls) demonstrating that the combination of methylated *AHANAK* and *STAP1* showed good diagnostic performance for HBV-related hepatopathy, whereas *STAP1* hypermethylation emerged as an unfavorable prognostic biomarker for HCC patients [197]. Similarly, Xing et al. demonstrated that the aberrant methylation of *APC* could serve as a valuable epigenetic biomarker for both the diagnosis and prognosis of HCC patients [201]. In particular, the authors reported that increased methylation levels of *APC* not only differentiated HCC patients from healthy individuals but also predicted clinical outcomes, since higher methylation levels were detected in stage III/IV compared to stage I/II HCC patients [201].

#### Thyroid cancer

THCA can be classified into four distinct subtypes: papillary thyroid carcinoma (PTC), follicular thyroid carcinoma (FTC), medullary thyroid carcinoma (MTC), and anaplastic thyroid carcinoma (ATC). Currently, fine needle aspiration (FNA) biopsy is the gold standard method for PTC diagnosis. However, the FNA procedure has several limitations, including false negatives and indeterminate results. Additionally, the MTC subtype presents significant diagnostic challenges due to high variability in serum calcitonin levels [258, 259]. Consequently, recent research has focused on identifying novel THCA biomarkers to address these diagnostic limitations.

A search of the PubMed public database, based on the adopted criteria, identified nine preclinical studies that validated the significance of DNA methylation as a THCA-specific biomarker, primarily in tissue and plasma samples (Table 1). Regarding diagnostic potential, Li and colleagues evaluated the role of *RUNX family transcription factor 1* (*RUNX1*) methylation in PTC tumorigenesis, invasiveness, and lymph node metastasis [204]. Their findings revealed that a specific CpG site of the *RUNX1* gene (cg017255383), mapped into the 5’ untranslated region (5’UTR), was significantly hypomethylated in PTC tissues compared to benign thyroid nodules (SE: 75%; SP: 89%; AUC: 0.81) and inversely correlated with *RUNX1* mRNA levels [204]. Another recent study validated the diagnostic potential of *thyroid-stimulating hormone receptor* (*TSHR*) methylation by analyzing plasma samples from a cohort of 46 PTC patients and 57 healthy controls [206]. Specifically, Kazlauskiene and colleagues observed higher *TSHR* methylation levels in PTC patients compared to controls, while strong demethylation of *TSHR* was detected in post-thyroidectomy samples. Furthermore, *TSHR* methylation was positively correlated with tumor size and lymphovascular invasion, highlighting its potential as an epigenetic biomarker for THCA diagnosis and management [206]. In this context, Wang and colleagues investigated the diagnostic potential of a panel of DMRs associated with immune response-related genes, including *fucosyltransferase 4* (*FUT4*), *suppressor of cytokine signaling 3* (*SOCS3*), *interferon-induced transmembrane protein 1* (*IFITM1*), *CD40 molecule* (*CD40*), and *solute carrier family 7 member 8* (*SLC7A8*) [208]. Using blood leukocyte samples, the bisulfite sequencing revealed that hypomethylation of these DMRs had high diagnostic accuracy (SE: 83.33%; SP: 90.91; AUC: 0.858) for distinguishing malignant thyroid nodules from benign ones in the Chinese population [208].

Regarding the clinical significance of DNA methylation as a prognostic biomarker for THCA, Xiao et al. focused on *activating transcription factor 3* (*ATF3*), a gene whose role in tumor initiation and progression remains controversial [209]. The authors demonstrated that promoter hypermethylation of *ATF3* was associated with decreased PFS of PTC patients due to gene silencing, which in turn induced tumor progression by dysregulating prognosis-related genes [209]. Another noteworthy study retrospectively explored the role of *solute carrier family 5 member 8* (*SLC5A8*) methylation in predicting the prognosis of FTC patients [210]. Interestingly, FTC patients with partially or hypermethylated *SLC5A8* exhibited higher recurrence rates during a 5-year follow-up compared to those with hypomethylated *SLC5A8*, suggesting that methylation analysis of this gene could serve as a valuable prognostic tool for predicting clinical outcomes in FTC patients [210]. Recently, Shen and colleagues identified and validated a mutually exclusive DNA methylation signature to predict clinical outcomes in MTP patients and guide treatment decisions [212]. Specifically, DNA methylation profiling of the *Neuronatin* (*NNAT*) gene revealed that promoter hypermethylation negatively impacted mRNA expression levels in MTP patients with reduced OS, highlighting its potential as a valuable predictive and prognostic biomarker for this THCA subtype [212].

#### Cervical cancer

CC may be classified into several histological subtypes, each characterized by distinct etiology, screening effectiveness, and prognosis. Among these, cervical squamous cell carcinoma and endocervical adenocarcinoma (CESC) is the most common subtype, accounting for 80% of all cases, followed by adenosquamous carcinoma (ASC), small cell neuroendocrine carcinoma (SCNEC), clear cell adenocarcinoma of the cervix (CCAC), and other less common subtypes [260]. Over the years, numerous studies have highlighted the critical role of DNA methylation in the progression from precancerous conditions, such as cervical intraepithelial neoplasia (CIN), to CESC [261–263]. Despite the importance of these findings, the clinical application of DNA methylation hotspots as adjunct diagnostic and prognostic biomarkers for CC remains limited due to SE issues and a lack of SP, which can lead to false positives.

Among the 138 preclinical studies listed in Table 1, 17 scientific publications reported the clinical significance of DNA methylation in CC. Interestingly, the *paired box 1* (*PAX1*), *growth hormone secretagogue receptor* (*GHSR*), *somatostatin* (*SST*), *zinc finger of the cerebellum 1* (*ZIC1*), *family with sequence similarity 19 (chemokine (C–C motif)-like) member A4* (*FAM19A4*), and *achaete-scute family bHLH transcription factor 1* (*ASCL1*) genes have been investigated as both diagnostic and prognostic biomarkers (Table 1). In this context, although *SEPT9* aberrant methylation has been widely proposed as a valuable epigenetic biomarker for various tumor types, Bu and colleagues recently explored its diagnostic potential for CC [215]. Notably, their findings demonstrated that the detection of methylated *SEPT9* exhibited high diagnostic accuracy for the early diagnosis of cervical precancerous lesions. Additionally, increased *SEPT9* methylation levels, particularly in plasma samples from CC patients with pelvic nodal metastasis, underscored its potential application as a non-invasive prognostic biomarker [215]. Interestingly, Wever et al. proposed a panel of nine hypermethylated genes, including *adenylate cyclase-activating polypeptide 1* (*ADCYAP1*), *basic helix–loop–helix family member E22* (*BHLHE22*), *cadherin 13* (*CDH13*), *CDO1*, *galanin receptor 1* (*GALR1*), *GHSR*, *heart and neural crest derivatives expressed 2* (*HAND2*), *SST*, and *ZIC1*, that could represent a promising diagnostic marker (SE: 90%; SP: 90%; AUC: 0.83) for stage I CC patients when detected in urine, cervicovaginal self-samples, and cervical scrapes [218]. El-Zein and collaborators analyzed the methylation status of a panel of four genes in a validation set of 373 cervical scraping samples (102 CC, 53 CIN3, 61 CIN2, 57 CIN1, and 100 healthy subjects) [221]. Using bisulfite-based NGS analysis, the authors revealed that increased DNA methylation of *carbonic anhydrase 10* (*CA10*), *dipeptidyl peptidase like 10* (*DPP10*), *formin 2* (*FMN2*), and *hyaluronan synthase 1* (*HAS1*), both individually and in combination, accurately discriminated precancerous lesions (CIN1, CIN2, and CIN3) and CC patients from healthy controls (SE: 84.3%; SP: 95%; AUC: 0.9337), with highest methylation levels observed in CC patients [221].

Contrarily to the diagnostic studies, few preclinical studies have recently investigated the prognostic potential of DNA methylation hotspots. Although *PAX1* methylation analysis has been described as a valuable diagnostic tool for CC screening, Li and colleagues also explored its potential in predicting the clinical outcomes for CC patients [224]. Notably, their findings revealed that *PAX1* promoter hypomethylation led to the overexpression of the encoded protein, which in turn increased resistance to therapy, suggesting that aberrant methylation of *PAX1* may also serve as a reliable prognostic biomarker [224]. Among the most relevant studies meeting the selection criteria, Gilham and colleagues recently demonstrated that methylation analysis of *erythrocyte membrane protein band 4.1 like 3* (*EPB41L3*), when combined with HPV genotyping, significantly enhanced the predictive value, as *EPB41L3* hypermethylation and HPV + status were directly associated with disease progression to invasive CC [226]. In this context, Dick et al. recently identified a highly sensitive gene-related methylation signature for predicting the risk of recurrent CIN and CC [228]. Specifically, their study showed that methylation levels of *ASCL1*, *LIM homeobox protein* (*LHX*), and *FAM19A4* were significantly increased in women with recurrent CIN2/3, highlighting the prognostic value of these methylated genes for post-treatment follow-up [228].

#### Bladder cancer

The gold standard diagnostic procedures for BLCA detection include cystoscopy, urine cytology, and tissue biopsy. Although cystoscopy and urine cytology exhibit high SP, their SE is limited, particularly for low-grade tumors, often resulting in overdiagnosis and unnecessary invasive procedures [264]. In recent years, many studies have focused on the in silico identification of methylation-based biomarkers as non-invasive tests for BLCA, followed by validation on various biological matrices from cancer patients and healthy controls. As summarized in Table 1, 10 preclinical studies have evaluated the diagnostic significance of DNA methylation in both urine and tissue samples, whereas only six studies have investigated its prognostic potential.

Among the most significant studies, Oh et al. conducted a case–control study involving 175 BLCA patients and 143 healthy individuals to validate the diagnostic potential of a urine-based test analyzing *Proenkephalin* (*PENK*) methylation status [233]. Using the qMS-PCR, the authors reported that *PENK* hypermethylation effectively detected primary BLCA in patients with hematuria, with performance metrics surpassing standard methods (SE: 86.9%; SP: 91.6%; AUC: 0.892) [233]. Another recent study focused on the analysis of *death-associated protein kinase* (*DAPK*) methylation status in urinary sediment to enhance the early detection of recurrent BLCA [236]. Notably, Wang and colleagues highlighted that the detection of methylated *DAPK*, combined with B ultrasound, offered significant diagnostic value (SE: 92.86; SP: 91.63%; AUC: 0.922) [236]. Starting from seven potential epigenetic biomarkers, Zhang et al. also developed a diagnostic tool based on the methylation analysis of *neuritin 1* (*NRN1*) in urine samples from BLCA patients [239]. Interestingly, the study revealed that *NRN1* hypermethylation (chr6:6,004,463–6,004,464), in conjunction with point mutations in *telomerase reverse transcriptase* (*TERT*) and *fibroblast growth factor receptor 3* (*FGFR3*) (C228T and p.S249C, respectively), exhibited superior diagnostic performance in detecting BLCA compared to conventional methods [239].

Recent studies have also evaluated the clinical utility of DNA methylation hotspots as prognostic biomarkers for BLCA. For instance, Chen and colleagues identified a specific CpG site (cg16145324) located in the intragenic region of *mitogen-activated protein kinase 14* (*MAPK14*), whose hypomethylation was strongly associated with reduced OS in BLCA patients [241]. In a recent prospective study involving 186 non-muscle-invasive bladder cancer (NMIBC) patients, Han et al. evaluated the prognostic significance of *PENK* methylation [243]. Although *PENK* aberrant methylation has been associated with BLCA tumorigenesis, the study revealed that NMIBC patients with *PENK* hypermethylation exhibited reduced recurrence-free survival rates, underscoring its clinical value also as a prognostic biomarker for follow-up care of BLCA patients [243]. Furthermore, Monteiro-Reis et al. investigated the DNA methylation status of *vimentin* (*VIM*), demonstrating that its methylation levels increased during BLCA initiation and development [244]. However, this alteration was identified as a passenger epigenetic event, as *VIM* promoter hypomethylation and mRNA overexpression were linked to the epithelial-to-mesenchymal transition (EMT) of cancer cells, contributing to aggressiveness and disease progression [244].

#### Non-Hodgkin Lymphoma

Currently, the gold standard method for the diagnosis of NHL involves a combination of lymph node or tissue biopsy, comprehensive histological analysis, and diagnostic imaging strategies. However, the main diagnostic pitfalls are related to the histopathological complexity of NHL subtypes and the presence of coexisting infections, which can significantly affect the accuracy of diagnosis [265]. Among NHL subtypes, nasal natural killer/T-cell lymphoma (NKTCL) is a rare and aggressive form of lymphoma universally associated with Epstein–Barr virus (EBV), which often complicates its diagnosis due to similarities with nasopharyngeal cancer (NPC) [266]. Therefore, the identification of novel cancer-specific biomarkers for early-stage diagnosis, as well as the validation of predictive biomarkers for treatment response and resistance, could greatly improve the clinical outcomes for NHL patients.

As shown in Table 1, few recent studies have explored the diagnostic potential of aberrant DNA methylation in NHL compared to other tumor types, highlighting the need of a deeper knowledge of the relationship between epigenetic mechanisms and NHL initiation. In particular, Tian and colleagues examined ctDNA methylation profiles in patients with extranodal natural killer/T-cell lymphoma (ENKTCL) and healthy controls (*N* = 594 and 734, respectively) to identify specific methylation patterns associated with this malignancy [246]. The authors discovered a seven-methylation marker signature with high diagnostic accuracy (SE: 95.3%; SP: 95.8%; AUC: 0.994), where increased methylation levels enabled the differentiation between ENKTCL patients and healthy individuals [246]. Furthermore, Tang et al. focused on EBV methylation profiling by analyzing EBV CpG sites mapped into the *BamHI-A rightward frame 1-like protein 2* (*BILF2*) gene to address challenges in the early diagnosis of NKTCL [247]. Using qMS-PCR for the analysis of nasopharyngeal brushing samples, the study revealed distinct methylation patterns among the considered groups. Specifically, *BILF2* methylation levels were significantly reduced in NKTCL compared to NPC patients, offering a promising diagnostic biomarker to differentiate between these two malignancies (SE: 98.81%; SP: 76.92%; AUC: 0.9801) [247].

Similar to diagnosis, the prognostic value of DNA methylation for NHL has been also investigated in a limited number of preclinical studies in the last year. Notably, Wu and colleagues explored the predictive significance of *N-myc downstream regulated gene 2* (*NDRG2*) in patients with diffuse large B-cell lymphoma (DLBC), one of the most common types of NHL [248]. Interestingly, promoter demethylation of *NDRG2* was inversely correlated with mRNA expression levels and negatively impacted on cancer cell proliferation and survival by inhibiting the *MYC proto-oncogene, BHLH transcription factor* (*MYC*) and *Myc-interacting zinc finger protein 1* (*MIZ-1*) pathways. Survival analysis further confirmed the relationship between *NDRG2* expression levels and DNA methylation status, suggesting that *NDRG2* promoter hypomethylation could serve as a favorable prognostic biomarker for DLBCL patients [248]. Another recent study focused on multi-omic analyses and methylation sequencing to characterize molecular and clinical subtypes of primary central nervous system lymphoma (PCNSL), a rare and aggressive form of NHL [249]. Among the identified molecular subtypes, the CS2 group showed a higher number of hypermethylated CpG hotspots compared to the other groups. In particular, methylation enrichment within the promoter regions of the *B-cell CLL/lymphoma 11A* (*BCL11A*), *B-cell CLL/lymphoma 6* (*BCL6*), and *IRF4* genes exhibited a negative prognostic impact on OS in PCNSL patients [249]. Furthermore, Nowialis and colleagues characterized the epigenetic aberrations associated with poor clinical outcomes in peripheral T-cell lymphoma (PTCL), an aggressive T-cell malignancy with limited treatment options [250]. Genome-wide methylation profiling of PTCL tissues showed a significant number of DNA methylation patterns affecting key genes involved in the regulation of cancer cell proliferation and survival. Notably, *thyroid hormone receptor interactor 13* (*TRIP13*) was identified as a driver gene, whose promoter hypomethylation was strictly correlated with reduced OS, suggesting that epigenetic targeting of *TRIP13* could represent a valuable treatment strategy for PTCL [250].

## Clinical studies on DNA methylation-based biomarkers in cancer diagnosis and prognosis

Over the last few decades, an increasing number of observational clinical studies have been initiated to validate DNA methylation hotspots as cancer-specific biomarkers in real-world clinical settings. The validation of these DNA methylation hallmarks could significantly enhance personalized cancer care, allowing for earlier detection, better risk stratification, and tailored therapeutic interventions. In this context, a comprehensive analysis of clinical studies deposited on https://clinicaltrials.gov/ (last accessed October 16, 2024) was conducted to report the most relevant investigations on the topic. To this end, the keywords "Cancer," "Methylation", and either "Diagnosis" or "Prognosis" were used in the search. Clinical studies were then selected according to the following criteria: (i) the number of participants in the study had to be greater than 50 subjects for diagnostic studies and 20 for prognostic studies; (ii) the clinical study, for both diagnosis and prognosis, had to investigate a specific gene-related DNA methylation target and not the whole methylation status. The adopted approach allowed to identify a total of 40 clinical trials, of which 27 studies (67.5%) investigated the diagnostic potential of DNA methylation hotspots, while 13 studies (32.5%) focused on the methylation levels of cancer-related genes as prognostic biomarkers (Table 2). Among the selected clinical trials, a detailed description of the most relevant and completed studies is also provided below.

**Table 2:** Clinical studies on DNA methylation as a diagnostic and prognostic cancer-related biomarker (deposited on https://clinicaltrials.gov/—last accessed October 16, 2024)

Study ID	Disease	Methodology	Biomarker	Clinical relevance	Size	Status	References
NCT00855348	CRC	Epi proColon	*SEPT9*	Diagnosis	7,929	C	[267, 268]
NCT01511653	CRC	qMS-PCR	*VIM*, *BCAT1*, *IKZF1*,* LINE1*	Diagnosis	13,000	C	N/A
NCT01397747	CRC	qMS-PCR	*NDRG4*,* BMP3*	Diagnosis	12,776	C	[269]
NCT02476682	CRC	Multi-plexed RT-PCR	*BCAT1*, *IKZF1*,* ACTB*	Diagnosis	205	C	N/A
NCT05508503	CRC	ctDNA dual-target test kit	*NTMT1*,* MAP3K14-AS1*	Diagnosis	1,378	C	[270, 271]
NCT04304131	CRC	RT-PCR	*SDC2*	Diagnosis	1,210	C	[272]
NCT02540850	CRC	*SEPT9* gene meylation	*SEPT9*	Diagnosis	1,031	C	[273]
NCT04287335	CRC	qMS-PCR	*BMP3*,* NDRG4*	Diagnosis	4,758	C	N/A
NCT01270360	CRC	GoldenGate Methylation Cancer Panel I	*PENK*, *NPY*,* WIF1*	Diagnosis	502	C	[274]
NCT05996458	GC	qMS-PCR	*RNF180*, *SEPT9*	Diagnosis	84,000	R	N/A
NCT05991947	GC	NGS	*APC*, *BRAF*, *EGFR*, *CDKN2A*, *AKT1*, *CTNNB1*, *FBXW7*, *FGFR2*, *GNAS*, *HRAS*, *KRAS*, *NRAS*, *PIK3CA*, *RNF43*, *PPP2R1A*, *PTEN*, *TP53*,* TOP2A*	Diagnosis	1,100	R	N/A
NCT04994197	BLCA	DNA Methylation-Lightning™ Kit	*ONECUT2*	Diagnosis	970	R	[275]
NCT05362539	BLCA	qMS-PCR	*GATA4*, *p16*, *p14*, *APC*, *CDH1*, *CD99*	Diagnosis	246	C	N/A
NCT05220189	BLCA	EarlyTect® Bladder Cancer test	*PENK*	Diagnosis	1,549	R	[276]
NCT04568512	CCA	qMS-PCR	*HOXA1*, *NEUROG1*	Diagnosis	67	C	[277]
NCT00835341	OC	qMS-PCR	*p16*	Diagnosis	93	C	[278]
NCT01695018	OC	MethyLight	*p16*	Diagnosis	180	C	[279]
NCT01945697	OC	qMS-PCR	*ZNF582*,* PX1*	Diagnosis	267	C	[280]
NCT05573217	HCC	qMS-PCR	*PIVKA-II*	Diagnosis	180	US	N/A
NCT03804593	HCC	HCCBloodTest	*SEPT9*	Diagnosis	175	C	[281]
NCT03483922	HCC	NGS	*AHNAK*, *STAP1*	Diagnosis	403	C	N/A
NCT05801263	OV	*CDO1/HOXA9* methylation assay	*CDO1*, *HOXA9*	Diagnosis	5,000	R	N/A
NCT03311152	HCC	Epi proColon 2.0 CE	*SEPT9*	Diagnosis	530	US	N/A
NCT06367049	NPC	qMS-PCR	*H4C6*, *SEPT9*, *RASSF1A*	Diagnosis	470	C	N/A
NCT05680077	EC	Fluorescence PCR	*KCNA3*, *OTOP2*	Diagnosis	1,116	C	N/A
NCT04321499	LUAD	RT-PCR	*SHOX2*,* PTGER4*	Diagnosis	70	C	[282]
NCT00340717	PRAD	qMS-PCR	*GSTPI*, *CD44*, *Cav-1*, *ANXA2*	Diagnosis	100	C	N/A
NCT00509821	GBM	Pyrosequencing	*MGMT*	Prognosis	60	C	N/A
NCT00897819	CRC	Pyrosequencing	*APBA1*, *APBA3*, *p14*, *p16*	Prognosis	350	C	N/A
NCT02786602	CRC	Pyrosequencing	*LRP1*	Prognosis	345	C	N/A
NCT04893356	SARC	qMS-PCR	*MGMT*	Prognosis	75	A, NR	[283]
NCT02022995	CRC	qMS-PCR	*EGFR*	Prognosis	180	C	N/A
NCT01139944	NSCLC	qMS-PCR	*p16*, *CDKN2A*, *APC*, *RASSF1A*, *DAPK1*, *CDH13*	Prognosis	99	C	N/A
NCT04177316	HCC	Pyrosequencing	*VTRNA2-1*	Prognosis	92	C	[284]
NCT04830618	GC	quantitative MethyLight assay	*MOS*	Prognosis	300	C	[285]
NCT02159339	GC	MethyLight	*E-cad*, *GFRA1*, *p16*, *SRF*, *ZNF382*	Prognosis	198	C	[286]
NCT01715233	GC	qMS-PCR	*CHFR*	Prognosis	27	C	N/A
NCT04155242	EC	MethyLight	*ZNF345*, *ZNF569*, *TFPI2*	Prognosis	147	R	N/A
NCT03542097	EWS	qMS-PCR	*MGMT*	Prognosis	82	C	N/A
NCT02688491	KIRC	qMS-PCR	*PITX1*, *FOXE3*, *RIN1*, *TWF2*,* EHBP1L1*	Prognosis	300	NR	N/A

As reported in Table 2, most of the selected clinical studies focused on CRC (*N* = 12), followed by GC (*N* = 5) and HCC (*N* = 5). Regarding the most investigated genes, *SEPT9* and *cyclin-dependent kinase inhibitor 2A* (*p16INK4a*), also known as *p16*, were tested independently in six different clinical trials. Notably, the aberrant methylation of *SEPT9* was evaluated in CRC, GC, HCC, and NPC patients, whereas *p16* methylation was estimated in CRC, GC, BLCA, oral cancer (OC), and non-small cell lung cancer (NSCLC) patients. Furthermore, the DNA methylation status of the *APC* and *O-6-methylguanine-DNA methyltransferase* (*MGMT*) genes was separately explored in three studies. Specifically, the methylation analysis was performed on GC, BLCA, and NSCLC patients for *APC,* whereas GBM, sarcoma (SARC), and Ewing sarcoma (EWS) only for *MGMT*.

Among the CRC clinical trials, the study NCT00855348 validated the efficacy of Epi proColon for the diagnosis of asymptomatic CRC. Of note, Epi proColon received the US Food and Drug Administration (FDA) approval as a clinic in vitro diagnostic assay for the detection of methylated *SEPT9* in CRC plasma samples [267, 268]. Subsequently, Wu and colleagues proposed a simplified *SEPT9* assay for CRC detection, based on a single PCR reaction and an automated procedure for plasma extraction [273]. The proposed assay was validated through an observational study in a cohort of 1,031 CRC patients (NCT02540850), demonstrating that the SE of the newly *SEPT9* assay was enhanced when combined with carcinoembryonic antigen testing (SE: 86.4%) or fecal immunochemical testing (SE: 94.4%) [273]. In addition to CRC, aberrant *SEPT9* methylation was also proposed as a diagnostic biomarker for other cancer types. Notably, the study NCT03804593 demonstrated the association between *SEPT9* hypermethylation and HCC by using the HCCBloodTest, an in vitro assay for the detection of *SEPT9* methylation in plasma samples. Specifically, the HCCBloodTest showed a SE of 76.7% and SP of 64.1%, providing a valuable diagnostic tool for HCC [281].

As reported in recent preclinical studies, gut microbiota dysbiosis may play a pivotal role in CRC carcinogenesis by altering the DNA methylation status of both oncogenes and tumor suppressor genes [287–289]. In this context, the study NCT01270360 explored the potential association between gut microbiota dysbiosis and aberrant DNA methylation in CRC patients [274]. In particular, the study reported that microbiota dysbiosis in CRC patients was closely related to promoter hypermethylation of the *WNT Inhibitory Factor 1* (*WIF1*), *PENK*, and *Neuropeptide Y* (*NPY*) genes. Consequently, the authors established a cumulative methylation index (CMI), which was proposed as a potential biomarker for CRC diagnosis [274].

Aberrant promoter methylation of the *p16* gene is another epigenetic phenomenon observed in various cancer types. In this context, two distinct clinical trials (NCT00835341 and NCT01695018) investigated the correlation between *p16* promoter hypermethylation and OC initiation. In particular, Cao and colleagues conducted a prospective cohort study (NCT00835341) to evaluate the predictive value of *p16* methylation in patients diagnosed with oral epithelial dysplasia, demonstrating that the progression rates to OC were significantly higher in patients with *p16* promoter hypermethylation compared to those with unmethylated *p16* [278]. Similar findings were reported by the observational study NCT01695018, highlighting the potential application of *p16* methylation as an epigenetic hallmark of OC development [279].

Regarding *MGMT*, the retrospective study NCT04893356 explored the prognostic significance of its promoter methylation on clinical outcomes of dacarbazine-treated SARC patients. Notably, the study demonstrated that SARC patients with unmethylated *MGMT* had better PFS and a higher disease control rate compared to those with promoter hypermethylation [283]. Conversely, no published results were available yet for the remaining two clinical trials evaluating the prognostic relevance of *MGMT* methylation in glioblastoma and Ewing sarcoma (NCT00509821 and NCT03542097, respectively) (Table 2).

Compared to clinical trials on DNA methylation as a diagnostic biomarker, the limited number of clinical studies investigating DNA methylation as a prognostic marker indicates that the role of DNA methylation in cancer prognosis remains insufficiently explored. Besides the aforementioned prognostic study on *MGMT,* only three studies assessing the prognostic relevance of DNA methylation had related publications (Table 2). In particular, the study NCT04177316 investigated the prognostic value of *Vault RNA 2–1* (*VTRNA2-1*), a non-coding RNA transcript epigenetically regulated through 18 CpG dinucleotides within its promoter region [284]. Notably, Yu et al. conducted an observational study on 92 HCC patients, demonstrating that the methylation levels of the *VTRNA2-1* promoter were higher in tumor tissues than adjacent normal tissues and significantly correlated with unfavorable clinical outcomes in HCC patients [284]. Interestingly, the prospective study NCT04830618 evaluated the prognostic relevance of *Mos proto-oncogene* (*MOS*) methylation in a cohort of patients who underwent chirurgical resection for gastric dysplasia or early GC. Interestingly, CG patients with higher *MOS* methylation levels had an increased risk of metachronous recurrence compared to *MOS* low-methylation group [285]. Another observational study (NCT02159339) evaluated the efficacy of a panel of DNA methylation markers as a prognostic tool in a cohort of 198 GC patients [286]. In particular, the authors identified aberrant methylation of the *GDNF family receptor alpha 1* (*GFRA1*) and *zinc finger protein 382* (*ZNF382*) genes as the best combination in predicting GC metastasis, contributing to significantly improved management of GC patients in the context of perioperative chemotherapy [286].

Overall, the reported clinical trials underscore the growing importance of DNA methylation in cancer detection and management. Although some methylation-based tests have already been introduced into standard procedures, further research is needed to provide strong evidence of their impact on early diagnosis and personalized medicine. Moreover, technical and standardization challenges must be addresses to achieve their widespread clinical use.

## DNA methylation as a therapeutic target for cancer treatment

In recent years, the development of epidrugs (e.g., DNMT inhibitors, histone deacetylase [HDAC] inhibitors, histone methyltransferase [HMT] inhibitors) has garnered extensive attention as a promising therapeutic option for the treatment of both hematological malignancies and solid tumors [290–292].

**Fig. 3:**
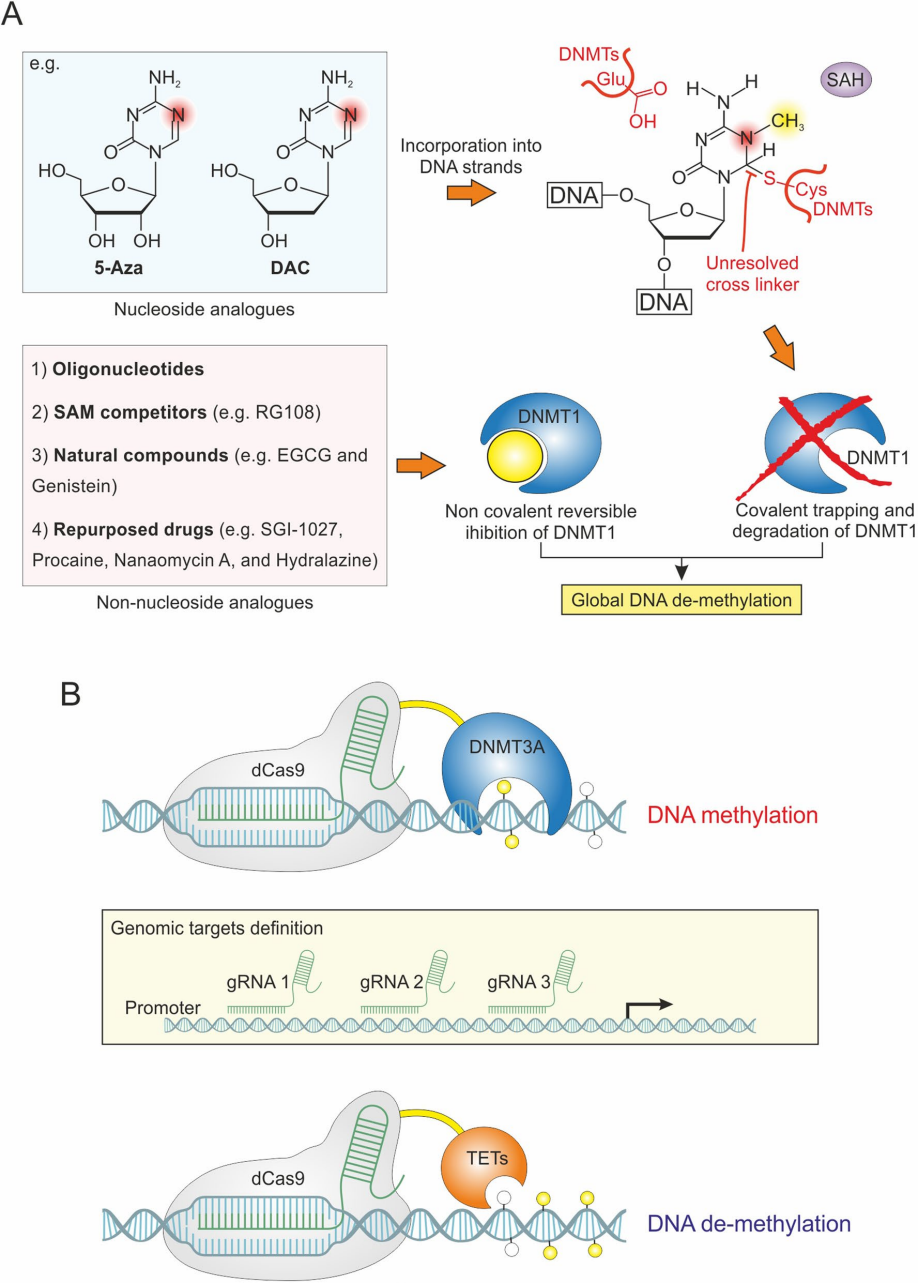
Targeting DNA methylation for epigenetic therapy. **A** Schematic representation of nucleoside and non-nucleoside analogs mechanism of action. **B** Schematic representation of epigenetic therapy based on the CRISPR-Cas9 system

Among epidrugs, DNMT inhibitors are classified into nucleoside and non-nucleoside analogs based on their mechanism of action. Nucleoside analogs, characterized by a modified cytosine ring that structurally resembles naturally occurring nucleosides, are incorporated into DNA during replication in place of cytosine. Notably, DNMTs recognize nucleoside analogs as natural substrates and catalyze the DNA methylation reaction by binding to DNA at the C-6 position of the cytosine ring. However, the covalent cross-linking cannot be resolved due to the presence of a nitrogen atom at the C-5 position, leading to DNMTs inactivation. Consequently, proteome-mediated degradation of DNMTs leads to DNA damage through the induction of double-strand breaks (DSBs) and the subsequent loss of methylation marks in daughter cells (Fig. 3A) [293, 294].

Interestingly, 5-azacytidine (5-Aza) (Vidaza®, Celgene Corporation) and 5-aza-2’-deoxycytidine, also known as decitabine (DAC) (Dacogen®, Otsuka America Pharmaceutical, Inc.), are the most extensively studied first-generation DNMT inhibitors. Specifically, 5-Aza affects the expression levels of DNMT1 and can be incorporated into both DNA and RNA, whereas DAC targets both DNMT1 and DNMT3A and is incorporated exclusively into DNA [295]. Currently, 5-Aza and DAC have been approved by the European Medicines Agency (EMA) and FDA for the treatment of hematological tumors, including acute myeloid leukemia (AML), chronic myeloid leukemia (CML), chronic myelomonocytic leukemia (CMML), and myelodysplastic syndrome (MDS) [296, 297]. Although both 5-Aza and DAC have significantly improved the management of hematological tumors, these first-generation hypomethylating agents are rapidly hydrolyzed in aqueous acidic or basic conditions, resulting in poor bioavailability and limited half-life. Additional limitations include a lack of target selectivity, as well as unfavorable pharmacodynamics and pharmacokinetics [298, 299]. In this field, various nucleoside analogs have been developed over the years to address the challenges associated with 5-Aza and DAC treatments. For instance, zebularine, a cytidine analog lacking the amino group at position four of the pyrimidine ring, exhibits high stability under acidic and neutral pH conditions. Moreover, it is less toxic compared to 5-Aza and DAC, enabling oral administration. Several studies highlighted that zebularine targets cancer cells by inhibiting cell growth and inducing apoptosis in HCC, CRC, BRCA, and head and neck cancer (HNCA) cell lines [300–303]. However, further investigations and clinical trials are necessary to confirm its effectiveness for the treatment of solid tumors. Other alternative DNMT inhibitors showing promising results in preclinical and clinical studies include 4′-thio-2′-deoxycytidine (TdCyd) and 5’-fluoro-2’-deoxycytidine (FdCyd). As reported by Thottassery and colleagues, TdCyd and FdCyd significantly affect DNMT1 activity and reduce cancer cell growth both in vitro and in vivo [304]. Furthermore, SGI-110, also known as guadecitabine, is a second-generation hypomethylating agent derived from DAC coupled with the dinucleotide analog deoxyguanosine, offering a prolonged half-life and enhanced activity. Recent studies have evaluated guadecitabine in combination with immunotherapy and anticancer drugs for the treatment of solid tumors, revealing its promising anticancer activity [305–308].

Besides the reported nucleoside analogs, numerous non-nucleoside analogs have been developed to counteract aberrant DNA methylation. These epidrugs are typically small-molecule DNMT inhibitors that directly affect catalytic sites, whose mechanism of action is unrelated to DNA incorporation. Typical non-nucleoside analogs include oligonucleotides, SAM competitors, natural compounds, and repurposed drugs with demethylating effects (Fig. 3A) [309–311]. Among these, SGI-1027, a quinoline-based compound, inhibits DNA methylation by targeting DNMT1, DNMT3A, and DNMT3B. In particular, it has been reported that SGI-1027 induces apoptosis in Huh7 and HeLa cells via the mitochondrial-mediated pathway [312, 313]. Similarly, the anesthetic procaine has been recently proposed as a non-nucleoside inhibitor with antitumor activity against GC cell lines [314]. Other repurposed drugs with high specificity for DNMT inhibition include the vasodilator hydralazine and the antibiotic nanaomycin A. Notably, hydralazine has been shown to reverse aberrant DNA methylation in leukemic T-cells, while nanaomycin A exhibits similar activity in neuroblastoma cells [315, 316]. Moreover, the SAM competitor N-phthaloy-L-tryptophan (RG108) has been identified as a valuable non-nucleoside analog with hypomethylating effects. Previous studies have demonstrated that RG108-induced DNA demethylation significantly reduces radioresistance in esophageal cancer cells and suppresses tumor growth in PRAD cell lines [317, 318]. Interestingly, recent research highlights the crucial role of natural compounds in reactivating tumor suppressor genes through DNMT inhibition. Among these, epigallocatechin-3-gallate (EGCG), a natural polyphenol commonly found in green tea, has demonstrated anticancer activity by targeting DNA hypermethylation and reactivating tumor suppressor genes in BRCA and HNCA [319, 320]. Furthermore, Romagnolo and colleagues have reported that the isoflavone genistein exhibits anticancer activity in *estrogen receptor alpha* (*ERα*)-negative BRCA cell lines, restoring the expression of *breast cancer 1 early onset* (*BRCA1*) and ERα via CpG demethylation [321].

In this field, epigenome editing has recently emerged as a novel approach for efficiently modulating gene expression by altering DNA methylation status of a specific target without changing the underlying DNA sequence. Notably, epigenome editing relies on designer editors comprising an Effector Domain (ED), derived from enzymes that influence epigenome (e.g., DNMTs, TETs, HATs, HDACs, transcriptional activators, and repressors), and a programmable DNA-binding domain (DBD) that guides the complex to the target site (e.g., zinc finger proteins [ZFPs], transcription activator-like effectors [TALEs]) [322, 323]. Besides ZFPs and TALEs, recent studies have shown that epigenetic therapy is also focusing on the clustered regularly interspaced short palindromic repeats (CRISPR)-Cas9 system [324–328]. The Cas9 endonuclease plays a crucial role in gene editing by cleaving the target sequence in the genome. Another key element of the CRISPR-Cas9 system is the single guide RNA (sgRNA), which comprises two parts: a short sequence designed to bind the target DNA, known as CRISPR RNA (crRNA), and a non-coding trans-activating crRNA required for Cas9-mediated cleavage. The crRNA sequence must be followed by a short DNA sequence of 2–6 bp, known as the protospacer adjacent motif (PAM), which is important for Cas9 compatibility. Briefly, the sgRNA drives Cas9 to cleave the target DNA at three nucleotides upstream of the PAM, generating DSBs that are then repaired by cell machinery based on the presence of a repair template. Notably, an exogenous donor DNA template is essential to achieve precise genome editing (Fig. 3B) [329–331].

In recent years, various strategies have been developed to modulate DNA methylation and, consequently, gene expression in cancer cells using the CRISPR-Cas9 system. For instance, recent studies have proposed a CRISPR-Cas9 based tool containing a deactivated Cas9 (dCas9) nuclease fused with a catalytic domain of DNMT and combined with co-expressed sgRNA, which achieves precise DNA methylation for specific targeted regions that are heritable across mitotic divisions in daughter cells [332, 333]. Another innovative strategy is based on the CRISPR-Cas9 demethylase tool, which consists of dCas9 and the catalytic domain of TET enzymes to selectively demethylate the targeted regions [334, 335]. In this context, various methods can be employed to ensure the efficient delivery of the CRISPR/Cas9 system, both in vitro and in vivo, depending on the specific requirements. Among these, both non-viral (e.g., cationic lipid-based vectors and cell-penetrating peptides) and viral (e.g., adeno-associated viral vectors, lentiviral vectors, and adenovirus vectors) vectors are commonly used to deliver plasmids or ribonucleoproteins expressing the nucleases, facilitating the delivery of the CRISPR/Cas9 system to target cells. Other approaches are based on physical methods, such as electroporation and microinjection [336, 337].

Despite the CRISPR-Cas9 system has revolutionized gene editing with its great potential in modulating gene expression and treating malignancies, several challenges remain unsolved for its clinical application. These include issues related to editing specificity and efficiency, delivery methods, and off-target effects. Therefore, further investigations are necessary to address these concerns and validate the use of the CRISPR-Cas9 system as an effective strategy for cancer treatment.

## Clinical studies on DNA methylation-targeted therapy in cancer care

As recorded on https://clinicaltrials.gov/, more than 1,000 clinical trials (41% Phase I, 49% Phase II, 9% Phase III, and 1% Phase IV) have investigated the effects of DNMT inhibitors over the years, either as monotherapy or in combination with existing therapeutic strategies for the treatment of various tumor types, particularly hematologic tumors (e.g., AML; MDS; CMML) and lymphomas (e.g., DLBC; Hodgkin lymphoma [HL]; NHL; peripheral T-cell lymphoma [PTCL]). In this context, ongoing and completed Phase III/IV clinical trials with an enrollment of more than 100 participants are summarized in Table 3 (https://clinicaltrials.gov/—last accessed October 16, 2024).

**Table 3:** Ongoing and completed Phase III/IV clinical trials (≥ 100 subjects) on DNA methylation-targeted therapy (listed on https://clinicaltrials.gov/—last accessed October 16, 2024)

Study ID	Disease	Treatment	Size	Phase	Status	References
NCT01074047	AML	5-Aza	488	III	C	[338, 339]
NCT00887068	AML, MDS	5-Aza	187	III	C	[340]
NCT04173533	AML, MDS	5-Aza	326	III	A, NR	N/A
NCT02319135	AML	5-Aza	289	III	C	N/A
NCT05175508	AML, MDS	5-Aza + ATRA	180	II/III	US	N/A
NCT05469737	MDS	5-Aza + BSC	230	II/III	A, NR	N/A
NCT01566695	MDS	5-Aza + BSC	216	III	C	[341]
NCT01757535	AML	5-Aza + BSC	472	III	C	[342–345]
NCT00071799	MDS	5-Aza + BSC	358	III	C	[346–350]
NCT03173248	AML, MDS	5-Aza + ivosidenib	146	III	A, NR	[351]
NCT05907057	AML	5-Aza + ivosidenib	245	III	R	N/A
NCT04256317	AML, MDS, CMML	5-Aza + cedazuridine	317	II/III	R	N/A
NCT04102020	AML	5-Aza + venetoclax	112	III	A, NR	N/A
NCT05404906	AML	5-Aza + venetoclax	124	II/III	R	N/A
NCT02993523	AML	5-Aza + venetoclax	443	III	A, NR	[352–355]
NCT04161885	AML	5-Aza + venetoclax	465	III	A, NR	N/A
NCT04401748	MDS	5-Aza + venetoclax	531	III	A, NR	N/A
NCT05939180	AML	5-Aza + venetoclax	116	II/III	R	N/A
NCT05079230	AML	5-Aza + venetoclax + magrolimab	378	III	T	N/A
NCT04778397	AML	5-Aza + venetoclax/magrolimab	258	III	T	N/A
NCT04799275	DLBC	5-Aza + chemotherapy	422	II/III	R	N/A
NCT05678933	PTCL	5-Aza + CHOP regimen	200	III	EBI	N/A
NCT03765541	AML	5-Aza + dexamethasone	142	III	R	N/A
NCT02158936	MDS	5-Aza + eltrombopag	356	III	T	[356]
NCT03745716	MDS	5-Aza + eprenetapopt	154	III	C	N/A
NCT02752035	AML	5-Aza + gilteritinib	183	III	A, NR	N/A
NCT03873311	AML, MDS, CMML	5-Aza + HAG regimen	114	IV	US	N/A
NCT05709093	MDS	5-Aza + lemzoparlimab	552	III	R	N/A
NCT04313881	MDS	5-Aza + magrolimab	539	III	T	N/A
NCT04090736	AML	5-Aza + pevonedistat	302	III	US	N/A
NCT03268954	AML, MDS, CMML	5-Aza + pevonedistat	454	III	A, NR	[357]
NCT03151408	AML	5-Aza + pracinostat	406	III	T	N/A
NCT04266301	MDS, CMML	5-Aza + sabatolimab	530	III	A, NR	N/A
NCT04797780	MDS	5-Aza + tamibarotene	550	III	R	N/A
NCT05075460	PTCL	5-Aza + tucidinostat + CHOP regimen	107	III	R	N/A
NCT02785900	AML	5-Aza/DAC + vadastuximab talirine	240	III	T	N/A
NCT03926624	AML	5-Aza/DAC + venetoclax	450	III	US	N/A
NCT06073730	AML	5-Aza/DAC + venetoclax	154	III	NYR	N/A
NCT05264883	AML	5-Aza/DAC + venetoclax + aclarubicin	170	III	R	N/A
NCT03257241	AML	DAC	582	III	US	N/A
NCT02072811	AML	DAC	400	III	US	N/A
NCT04292769	Solid tumors	DAC	200	III	US	N/A
NCT00043381	MDS	DAC	160	III	C	N/A
NCT00043134	MDS	DAC	220	III	US	N/A
NCT01751867	MDS	DAC	135	III	C	[358]
NCT00260832	AML	DAC	485	III	C	[359]
NCT02013102	MDS	DAC	240	IV	US	N/A
NCT02172872	AML	DAC	606	III	A, NR	N/A
NCT03026842	AML	DAC	180	IV	US	N/A
NCT04098653	Myeloid tumors	DAC + BUCY	196	II/III	US	N/A
NCT04123392	Myeloid tumors	DAC + BUCY	196	II/III	US	N/A
NCT03596892	AML, MLL	DAC + BUCY	122	II/III	US	N/A
NCT04510610	HL	DAC + camrelizumab	100	II/III	R	N/A
NCT02159820	OV	DAC + carboplatin + paclitaxel	500	II/III	US	N/A
NCT03306264	AML, MDS, CMML	DAC + cedazuridine	227	III	C	N/A
NCT03553537	PTCL	DAC + CHOP	100	III	US	N/A
NCT02085408	AML	DAC + cytarabine + clofarabine/daunorubicin	727	III	A, NR	N/A
NCT05449899	AML	DAC + G-CSF + BUCY/BF	232	II/III	R	N/A
NCT04087967	AML	DAC + HAAG regimen	162	III	US	N/A
NCT04446130	AML, T-ALL	DAC + HAAG regimen	100	III	US	N/A
NCT01303796	AML	DAC + sapacitabine	482	III	C	[360]
NCT02472145	AML	DAC + talacotuzumab	326	II/III	C	[361]
NCT02272478	AML, MDS	DAC + vosaroxin	1,600	II/III	US	N/A
NCT02348489	AML	SGI-110	815	III	C	N/A
NCT02920008	AML	SGI-110	302	III	C	N/A
NCT02907359	MDS, CMML	SGI-110	417	III	C	N/A

As shown in Table 3, a total of 66 Phase III/IV clinical trials were selected based on the aforementioned criteria. The selected trials included four studies investigating 5-Aza as a monotherapy and 35 evaluating its combination with other therapeutic agents. DAC was assessed as a monotherapy in 10 studies, while 18 focused on its use in combination therapies. Lastly, only three studies explored the therapeutic efficiency of SGI-110 in AML, MDS, and CMML. The main findings from clinical trials linked to scientific publications (*N* = 13) are briefly discussed below.

Among the Phase III/IV clinical trials reported in Table 3, the study NCT01074047 investigated the efficacy and safety of 5-Aza (Vidaza) (75 mg/m^2^ subcutaneously daily for 7 days for 28-day cycles) compared to conventional care regimen (CCR) (cytarabine 100–200 mg/m^2^ continuous intravenous infusion for 7 days + anthracycline IV for 3 days) in AML patients. Notably, the findings demonstrated that 5-Aza may be considered a valuable treatment option to improve OS in AML patients for whom intensive chemotherapy is not a viable strategy (OS 5-Aza group: 8.9 months; OS CCR group: 4.9 months) [338, 339]. Furthermore, the randomized controlled clinical trial NCT00887068 evaluated 5-Aza as a monotherapy maintenance treatment for AML and MDS patients (*N* = 187). Specifically, the treatment arm (32 mg/m^2^ subcutaneously for 5 days every 28 days for 12 cycles) showed higher relapse-free survival compared to the control group (2.07 years *vs* 1.28 years) [340]. In this context, various Phase III/IV clinical trials have explored the efficacy of 5-Aza treatment in combination with best supportive care (BSC) and other available anticancer strategies. For instance, the study NCT01566695 demonstrated that treatment with 300 mg CC-486 (oral azacytidine) + BSC for 21 days/28-day cycle significantly improved red blood cell transfusion independence (RBC IT) in MDS patients, further confirming the efficacy of 5-Aza in the treatment and management of hematologic tumors [341]. Similar findings were obtained in the NCT01757535 study, where the administration of CC-486 (300 mg for 14 days per 28-day cycle) + BSC as a maintenance treatment significantly enhanced both relapse-free survival and OS in de novo or secondary AML patients by reducing measurable residual disease (MRD) [342–345]. Interestingly, the NCT00071799 study reported that MDS patients treated with 5-Aza (75 mg/m^2^ subcutaneously) + BSC exhibited prolonged hematological responses and increased RBC IT compared to those receiving standard chemotherapy (cytarabine + anthracycline) + BSC (median OS: 24.5 months *vs* 16 months) [346–350]. Besides the aforementioned clinical trials, the study NCT03173248 also evaluated the effects of 5-Aza (75 mg/m^2^/day on days 1–7, or on days 1–5 and 8–9, of each 28-day cycle) in combination with ivosidenib (AG-120—500 mg orally, once daily), an inhibitor of mutant *isocitrate dehydrogenase 1* (*IDH1*), in *IDH1*-mutated AML patients. Notably, the experimental treatment significantly improved the OS of AML patients (24 months) and reduced common adverse events, such as neutropenia and infections, suggesting that 5-Aza-based therapies may provide meaningful clinical benefits in difficult-to-treat cancer patients [351]. Another Phase III clinical trial involving 443 AML patients (NCT02993523) demonstrated that the combination of 5-Aza (75 mg/m^2^) + Venetoclax (100 mg/200 mg/400 mg) was associated with high response rates, durable remissions, and significant improvements in OS among AML patients ineligible for standard chemotherapy [352–355]. Interestingly, the NCT02158936 study investigated the efficacy of 5-Aza (75 mg/m^2^ subcutaneously once daily for 7 days every 28 days) in combination with the thrombopoietin receptor agonist eltrombopag (50 mg, 100 mg, or 200 mg) as a first-line treatment strategy for MDS patients with thrombocytopenia. Notably, the combined therapy was related to reduced response rates and impaired platelet recovery, indicating that 5-Aza monotherapy was the preferred treatment option to increase PFS [356]. Conversely, the randomized Phase III clinical trial NCT03268954 demonstrated that high-risk MDS patients treated with 5-Aza (75 mg/m^2^ on days 1–5, 8/9 in 28-days cycles) + pevonedistat (20 mg/m^2^ intravenously on days 1–5 in 28-day cycles) experienced significantly higher event-free survival (EFS) and OS compared to those receiving 5-Aza monotherapy (median EFS: 19.2 months *vs* 15.6 months; median OS: 27.1 months *vs* 22.5 months) [357].

As shown in Table 3, recent clinical trials have reported promising evidence supporting DAC-based treatments of hematologic cancers. In particular, the study NCT01751867 highlighted that DAC (15 mg/m^2^ intravenously every 8 h for 3 consecutive days, 6-week treatment cycle) was effective and safe for the management of MDS patients, as demonstrated by improvements in hematologic parameters, median OS, and cytogenic response rates [358]. Similarly, another Phase III clinical trial (NCT00260832) showed that DAC monotherapy (20 mg/m^2^ intravenously for 5 consecutive days of each 28-day cycle) significantly improved response rates in elderly AML patients compared to cytarabine treatment (20 mg/m^2^) (odds ratio 2,751—95% CI: 1.487–5.091; *p* = 0.001) [359]. Furthermore, the study NCT01303796, a Phase III clinical trial involving 482 AML patients, evaluated the efficacy of DAC (20 mg/m^2^ once daily for 5 consecutive days every 8 weeks) + sapacitabine (300 mg twice daily on 3 consecutive days per week for 2 weeks every 8 weeks), an oral nucleoside analog. Notably, both complete remission (CR) rates and OS were significantly improved in AML patients with white blood cell counts < 10 × 10^9^/L who received the combined treatment compared to the control group (CR: 21% *vs* 8.6%; OS: 8 months *vs* 5.8 months) [360]. Interestingly, the NCT02472145 study, a randomized Phase II/III clinical trial enrolling 326 AML patients ineligible for intensive chemotherapy, reported that the combined therapy DAC (20 mg/m^2^ from day 1–5 of a 28-day cycle) + talacotuzumab (9 mg/kg on day 8 and 22 of 28-day cycle) significantly improved OS and enhanced the quality of life of AML patients [361].

Although most Phase III and IV clinical trials have focused on DNMT inhibitors for the treatment of hematological cancers and lymphoma, the targeting of DNA methylation has recently gained increasing attention as a promising therapeutic approach for various solid tumors [362–364]. Among the completed Phase I/II clinical trials on solid tumors, the pilot Phase I NCT02009436 study investigated the safety and efficacy of inhaled 5-Aza (15, 30, and 45 mg/m^2^ on days 1–5 and 15–19 of 28-day cycles) in patients with advanced NSCLC. Interestingly, inhaled 5-Aza was well tolerated, with no detectable systemic absorption, demonstrating great potential as an adjuvant treatment for reversing DNA methylation changes in the bronchial epithelium of LUAD patients at high risk of developing recurrent malignancy [365]. Similarly, the NCT00387465 study demonstrated that 5-Aza (30 mg/m^2^ subcutaneously) + entinostat (7 mg by mouth on days 3 and 10 of each cycle) significantly improved the PFI and OS of advanced NSCLC patients [366]. Furthermore, the combination therapies 5-Aza (100 mg daily subcutaneous injection on days 1–5 every 21 days) + Pembrolizumab (200 mg every 21 days) and 5-Aza (75 mg/m^2^ intravenous infusion over 30 min days 1—5 of each 3 weekly cycle) + docetaxel (75 mg/m^2^ intravenous infusion over 1 h on day 6 of each 3 weekly cycle) + prednisone (5 mg twice a day from day 1 to 21 of each cycle) showed clinical efficacy in the treatment of refractory CRC (NCT02260440) and PRAD (NCT00503984), respectively [367, 368]. Besides 5-Aza-based treatments, recent clinical trials have also focused on the clinical relevance of DAC in treating solid tumors. In particular, the NCT02316028 study demonstrated that hepatic arterial infusion of DAC (10, 15, and 20 mg/m^2^ 1 h for 5 days every 4 weeks) had no adverse events in patients with unresectable CRC and was associated with a significant upregulation of several tumor antigens, paving the way for further investigations on DAC + immunotherapy regimens [369]. Another Phase I/II clinical trial (NCT00879385) reported that DAC (45 mg/m^2^ over 2 h on day 1 and 15) + panitumumab (6 mg/kg over 1 h on day 8 and 22 every 28 days) showed clinical activity, as most CRC patients had stable disease or a partial response following the combined treatment [370]. Recently, SGI-110 has also been investigated as a valuable treatment option for advanced solid tumors. Interestingly, the NCT02901899 study demonstrated that the combined regimen of SGI-110 (30 mg/m^2^ subcutaneously on days 1–4 every 3 weeks) + pembrolizumab (200 mg on day 1 every 3 weeks) provided clinical benefit and antitumoral activity by reversing resistance to immune checkpoint inhibitors in patients with solid tumors [305]. Conversely, the randomized controlled trial NCT01966289 reported that SGI-110 (60 mg/m^2^ subcutaneously on days 1–5 every 28 days) combined with GVAX, an allogeneic CRC cell vaccine, and cyclophosphamide (200 mg/m^2^ intravenously on day 1) showed no significant immunologic activity in CRC patients [371]. Collectively, the Phase I/II clinical trials reported above highlight the potential clinical utility of DNMT inhibitors, both as monotherapy and in combination with standard therapeutic strategies, for the treatment of solid tumors. However, further advanced clinical investigations are necessary to provide strong evidence of their safety and efficacy in improving cancer patient outcomes.

## Conclusions and future perspectives

Among epigenetic modifications, DNA methylation is one of the most well-characterized mechanisms, playing a crucial role in both physiological and pathological conditions. Over the years, many studies have demonstrated the regulatory role of both promoter and intragenic DNA methylation status on the expression levels of cancer-related genes, underscoring the significance of aberrant DNA methylation in cancer initiation and progression. The detection of DNA methylation changes could be useful for improving early cancer diagnosis, distinguishing between different tumor subtypes, and predicting treatment response. Emerging advancements in DNA methylation-targeted therapy, including DNMT inhibitors and epigenome editing based on the innovative CRISPR-Cas9 system, pave the way for the development of innovative and effective therapeutic options for cancer treatment. However, the complexity of big data derived from DNA methylation profiling requires advanced technologies and a deeper understanding of their clinical application. In this context, deep analysis of methylome and computational approaches are essential for identifying DNA methylation hotspots as cancer biomarkers. Beyond single base-resolution hotspots, the aberrant methylation of large genomic regions has also emerged as a cancer-specific hallmark, whose detection provides high diagnostic and prognostic accuracy. Recent literature highlights that DNA methylation has attracted increasing attention for its potential application in clinical settings; however, heterogeneity among tumor types and even within the same tumor poses additional challenges for the identification of universal biomarkers. Moreover, the lack of standardized procedures for analyzing methylation hotspots significantly hampers their validation as routine biomarkers for cancer diagnosis and prognosis. Therefore, integrating DNA methylation data with other molecular markers could represent a valuable strategy to provide strong evidence for the clinical utility of DNA methylation. Despite DNMT inhibitors are widely used as a first-line treatment for hematological tumors, recent research has also explored these epidrugs as promising options for the treatment of solid tumors, particularly in combination with other therapeutic agents. Looking ahead, preclinical studies are investigating the safety and efficacy of epigenome editing, providing a valuable starting point for real-world applications of epigenetic-targeted strategies, leading to improved patient outcomes and the development of personalized medicine.

## Data Availability

The data reported in the manuscript are available from the corresponding author [LF] on reasonable request. The contributions presented in the study are publicly available. These data can be found at: https://pubmed.ncbi.nlm.nih.gov/; https://clinicaltrials.gov/;

## Abbreviations

↑: Increased
↓: Reduced
5-Aza: 5-Azacytidine
5-Aza-CdR: 5-Azacytidine-2′-deoxycytidine
5caC: 5-Carboxylcytosine
5fC: 5-Formylcytosine
5hmC: 5-Hydroxymethylcytosine
5mC: 5-Methylcytosine
5’UTR: 5’ Untranslated region
A: Active
A1AT: Alpha-1-ANTITRYPSIN
ABL1: ABL proto-oncogene 1 non-receptor tyrosine kinase
ACTB: Actin Beta
ADCYAP1: Adenylate cyclase-activating polypeptide 1
ADHFE1: Alcohol dehydrogenase iron containing 1
AFP: Alpha-fetoprotein
AGRN: Agrin
AHNAK: AHNAK nucleoprotein
AKT1: AKT serine/threonine kinase 1
AML: Acute myeloid leukemia
ANXA2: Annexin A2
APBA1: Amyloid beta precursor protein-binding family a member 1
APBA3: Amyloid beta precursor protein-binding family a member 3
APC: Adenomatous polyposis Coli
ASC: Adenosquamous carcinoma
ASCL1: Achaete-scute family BHLH transcription factor 1
ASTN1: Astrotactin 1
ATC: Anaplastic thyroid carcinoma
ATF3: Activating transcription factor 3
ATIC: Aminoimidazole-4-carboxamide ribonucleotide formyltransferase/IMP cyclohydrolase
ATRA: All-trans retinoic acid
AUC: Area under the curve
AXL4: AXL receptor tyrosine kinase
B3GNTL1: UDP-glucose ceramide glucosyltransferase like 1
BCAT1: Branched-chain amino acid transaminase 1
BCL11A: B-Cell CLL/lymphoma 11A
BCL6: B-Cell CLL/lymphoma 6
BCR: Biochemical recurrence
BER: Base excision repair
BF: Busulfan—fludarabine
BHLHE22: Basic helix–loop–helix family member E22
BILF2: BamHI-A rightward frame 1-like protein 2
BLCA: Bladder cancer
BMP3: Bone morphogenetic protein 3
BMP4: Bone morphogenetic protein 4
BNIP3: BCL2 interacting protein 3
BPH: Benign prostate hyperplasia
BRAF: B-raf proto-oncogene serine/threonine kinase
BRCA: Breast cancer
BRCA1: Breast cancer 1 early onset
BSC: Best supportive care
BSP: Bisulfite genomic sequencing PCR
BTC2: B-cell translocation gene 2
BTD: Biotinidase
BUCY: Busulfan–cyclophosphamide
BW: Bronchial washing
C: Completed
CA10: Carbonic anhydrase 10
CACNA1E: Calcium voltage-gated channel subunit alpha1 E
CAMK2N1: Calcium-/calmodulin-dependent protein kinase II inhibitor 1
Cav-1: Caveolin 1
CC: Cervical cancer
CC-486: Oral azacytidine
CCA: Cholangiocarcinoma
CCAC: Clear cell adenocarcinoma of the cervix
CCB: Cervical cytology brush
CCDC12: Coiled-coil domain containing 12
CCND2: Cyclin D2
CCR: Conventional care regimen
CD40: CD40 molecule
CD44: CD44 molecule
CD99: CD99 molecule
CDH1: Cadherin 1
CDH13: Cadherin 13
CDK6: Cyclin-dependent kinase 6
CDKL2: Cyclin-dependent kinase like 2
CDKN2A: Cyclin-dependent kinase inhibitor 2A
CDO1: Cysteine dioxygenase type 1
CEACAM5: CEA cell adhesion molecule 5
CELF6: CUGBP elav-like family member 6
CESC: Cervical squamous cell carcinoma and endocervical adenocarcinoma
cfDNA: Circulating-free DNA
CH_3_: Methyl group
CHFR: Checkpoint with forkhead and ring finger domains
CHOP: Cyclophosphamide—doxorubicin—vincristine—prednisone
CHST15: Carbohydrate sulfotransferase 15
CIN: Cervical intraepithelial neoplasia
CLEC14A: C-Type lectin domain family 14 member A
CMI: Cumulative methylation index
CML: Chronic myeloid leukemia
CMML: Chronic myelomonocytic leukemia
COL1A1: Collagen type I alpha 1 chain
COL1A2: Collagen type I alpha 2 chain
COL3A1: Collagen type III alpha 1 chain
COL25A1: Collagen type XXV alpha 1 chain
CpG: Cytosine–guanine dinucleotide
CR: Complete remission
CRC: Colorectal cancer
CRISPR: Clustered regularly interspaced short palindromic repeat
crRNA: CRISPR RNA
CSF: Cerebrospinal fluid
ctDNA: Circulating tumor DNA
CTNNB1: Catenin beta 1
CXCL12: C-X-C Motif chemokine ligand 12
Cys: Cysteine
DAC: Decitabine
DACH1: Dachshund family transcription factor 1
DAPK: Death-associated protein kinase
DAPK1: Death-associated protein kinase 1
DBD: DNA-binding domain
dCas9: Deactivated Cas9
DCP: Des-gamma-carboxyprothrombin
ddPCR: Droplet digital PCR
DFP: Disease-free progression
DFS: Disease-free survival
DLBC: Diffuse large B-cell lymphoma
DLGAP2: Discs large (drosophila) homolog-associated protein 2
DLK1: Delta-like non-canonical notch ligand 1
DLX1: Distal-less homeobox 1
DMC: Differential methylation at single cytosine
DML: Differentially methylated locus
DMR: Differentially methylated region
DNMT: DNA methyltransferase
DOK7: Docking protein 7
DPP6: Dipeptidyl peptidase like 6
DPP10: Dipeptidyl peptidase like 10
DSB: Double-strand break
E2F2: E2F transcription factor 2
EBI: Enrolling by Invitation
EBV: Epstein–Barr virus
EC: Endometrial cancer
E-cad: E-cadherin
ECE1: Endothelin-converting enzyme 1
ED: Effector domain
EFS: Event-free survival
EGCG: Epigallocatechin-3-gallate
EGD: Esophagogastroduodenoscopy
EGFR: Epidermal growth factor receptor
EHBP1L1: EH domain-binding protein 1 like 1
ELF5: E74-like ETS transcription factor 5
ELISA: Enzyme-linked immunosorbent assay
EMA: European medicines agency
EMT: Epithelial-to-mesenchymal transition
ENKTCL: Extranodal natural killer/T-cell lymphoma
EPB41L3: Erythrocyte membrane protein band 4.1 like 3
ERα: Estrogen receptor alpha
ERCC1: ERCC excision repair 1 endonuclease non-catalytic subunit
EWS: Ewing sarcoma
FAM19A4: Family with sequence similarity 19 (chemokine (C–C motif)-like) member A4
FBXW7: F-box and WD repeat domain containing 7
FDA: US Food and Drug Administration
FdCyd: 5’-Fluoro-2’-deoxycytidine
FFPE: Formalin fixed paraffin embedded
FGFR2: Fibroblast growth factor receptor 2
FGFR3: Fibroblast growth factor receptor 3
FLT1: Fms-related receptor tyrosine kinase 1
FMN2: Formin 2
FN1: Fibronectin 1
FNA: Fine needle aspiration
FOXA1: Forkhead box A1
FOXE3: Forkhead box E3
FOXF1: Forkhead box F1
FOXI2: Forkhead box I2
FOXO3: Forkhead box O3
FTC: Follicular thyroid carcinoma
FUT4: Fucosyltransferase 4
GALNT9: Polypeptide N-acetylgalactosaminyltransferase 9
GALR1: Galanin receptor 1
GATA4: GATA binding protein 4
GBM: Glioblastoma
GC: Gastric cancer
GCM2: Glial cells missing transcription factor 1
G-CSF: Granulocyte colony-stimulating factor
GFRA1: GDNF family receptor alpha 1
GHSR: Growth hormone secretagogue receptor
GLDC: Glycine dehydrogenase
Glu: Glutamic acid
GNAS: GNAS complex locus
GNB4: G protein subunit beta 4
GRAP2: GRB2-related adaptor protein 2
gRNA: Guide RNA
GSTM5: Glutathione S-transferase Mu 5
GSTP1: Glutathione S-transferase Pi 1
GSX1: Glycine decarboxylase 1
H4C6: H4 clustered histone 6
H19: H19 imprinted maternally expressed transcript
HAAG: Cytarabine—amsacrine—G-CSF**—**mitoxantrone
HAG: Homoharringtonine—cytarabine—G-CSF
HAND1: Heart and neural crest derivatives expressed 1
HAND2: Heart and neural crest derivatives expressed 2
HAS1: Hyaluronan synthase 1
HCC: Hepatocellular carcinoma
HDAC: Histone deacetylase
HIST1H4F: Histone cluster 1 H4 family member F
HL: Hodgkin lymphoma
HLX-AS1: H2.0-like homeobox antisense RNA 1
HMT: Histone methyltransferase
HNCA: Head and neck cancer
HOXA1: Homeobox A1
HOXA7: Homeobox A7
HOXA9: Homeobox A9
HOXB4: Homeobox B4
HOXD8: Homeobox D8
HOXD9: Homeobox D9
HPCE: High-performance capillary electrophoresis
HPLC: High-performance liquid chromatography
HRAS: HRas proto-oncogene GTPase
HSP90AB1: Heat shock protein 90 alpha family class B member 1
ICR: Imprinting control region
IDH1: Isocitrate dehydrogenase 1
IFITM1: Interferon-induced transmembrane protein 1
IKZF1: Ikaros family zinc finger 1
IL21R: Interleukin 21 receptor
IRF4: Interferon regulatory factor 4
IRF8: Interferon regulatory factor 8
IRX1: Iroquois homeobox 1
ITGA4: Integrin subunit alpha 4
ITPRIPL1: Inositol 145-trisphosphate receptor-interacting protein like 1
KCNA3: Potassium voltage-gated channel subfamily a member 3
KIRC: Kidney renal clear cell carcinoma
KLRD1: Killer cell lectin-like receptor D1
KLRK1: Killer cell lectin-like receptor K1
KRAS: KRAS proto-oncogene, GTPase
LBC: Liquid-based citology
LC–MS/MS: Liquid chromatography coupled with tandem mass spectrometry
LCP2: Lymphocyte cytosolic protein 2
LDCT: Low-dose computed tomography
LDHB: Lactate dehydrogenase B
LGALS3: Lectin galactoside-binding soluble 3
LHX: LIM homeobox protein
LHX8: LIM homeobox protein 8
LIHC: Liver cancer
LINC01594: Long intergenic non-protein-coding RNA 1594
LINC02115: Long intergenic non-protein-coding RNA 2115
LINE-1: Long interspersed nuclear element-1
LRP1: LDL receptor-related protein 1
LUAD: Lung cancer
LUMA: Luminometric methylation assay
MAL: Mal T-cell differentiation protein
MALAT1: Metastasis-associated lung adenocarcinoma transcript 1
MAP3K14-AS1: MAP3K14 antisense RNA 1
MAPK14: Mitogen-activated protein kinase 14
MBD: Methyl CpG-binding domain
MBDCap-Seq: Methyl-binding domain capture sequencing
mCRPC: Metastatic castration-resistant prostate cancer
mdMSP: Multiplex digital methylation‐specific PCR
MDS: Myelodysplastic syndromes
MeDIP: Methylcytosine-based DNA immunoprecipitation
MEM132D: Membrane protein 132D
METAP1D: Methionyl aminopeptidase type 1D mitochondrial
MethyQESD: Methylation quantification endonuclease-resistant DNA
MGMT: O-6-Methylguanine-DNA methyltransferase
MIR145: MicroRNA 145
MIR3973: MicroRNA 3973
MIR12123: MicroRNA 12123
MIZ-1: Myc-interacting zinc finger protein 1
MLH1: MutL homolog 1
MLL: Mixed lineage leukemia
MMP9: Matrix metallopeptidase 9
MOS: Mos proto-oncogene serine/threonine kinase
MRD: Measurable residual disease
MS-ddPCR: Methylation-sensitive droplet digital PCR
MS-dPCR: Methylation-specific chip-based digital PCR
MS-HRM: Methylation-sensitive high-resolution melting assay
MS-PCR: Methylation-sensitive PCR
MSRE-ddPCR: Methylation-sensitive restriction enzyme–droplet digital PCR
MSRE-qPCR: Methylation-sensitive restriction enzyme–quantitative PCR
MTC: Medullary thyroid carcinoma
MYC: MYC proto-oncogene BHLH transcription factor
MYO15B: Myosin XVB
N/A: Not Applicable
NAA10: N(Alpha)-acetyltransferase 10
NB: Nasopharyngeal brushing
NCAM2: Neural cell adhesion molecule 2
NCOA2: Nuclear receptor coactivator 2
ncRNA: Non-coding RNA
NDRG2: N-Myc downstream regulated gene 2
NDRG4: N-Myc downstream regulated gene 4
NEUROG1: Neurogenin 1
NGS: Next-generation sequencing
NHL: Non-hodgkin lymphoma
NKTCL: Nasal natural killer/T-cell lymphoma
NLRP2: NLR family pyrin domain containing 2
NLRP3: NLR family pyrin domain containing 3
NLRP7: NLR family pyrin domain containing 7
NMIBC: Non-muscle-invasive bladder cancer
NNAT: Neuronatin
NOVA1: NOVA alternative splicing regulator 1
NPC: Nasopharyngeal cancer
NPY: Neuropeptide Y
NR: Not recruiting
NRAS: NRAS Proto-Oncogene GTPase
NRN1: Neuritin 1
NSCLC: Non-small cell lung cancer
NTMT1: N-terminal Xaa-Pro-Lys N-methyltransferase 1
NXPH1: Neurexophilin 1
NYR: Not yet recruiting
OC: Oral cancer
ONECUT2: One cut homeobox 2
OS: Overall survival
OSR1: Odd-skipped related transcription factor 1
OTOP2: Otopetrin 2
OTX1: Orthodenticle homeobox 1
OV: Ovarian cancer
PAM: Protospacer adjacent motif
PAX1: Paired box 1
PAX3: Paired box 3
PAX5: Paired box 5
PBMC: Peripheral blood mononuclear cells
PCDHGA12: Protocadherin gamma subfamily A 12
PCDHGB7: Protocadherin gamma subfamily B 7
PCNSL: Primary central nervous system lymphoma
PCR: Polymerase chain reaction
PENK: Proenkephalin
PFS: Progression-free survival
PIK3CA: Phosphatidylinositol-4,5-bisphosphate 3-kinase catalytic subunit alpha
PITX1: Paired like homeodomain 1
PIVKA-II: Prothrombin induced by vitamin K absence-II
PLXNA4: Plexin A4
Pol II RNA: Polymerase II
PPP2R1A: Protein phosphatase 2 scaffold subunit aalpha
PPP2R5C: Protein phosphatase 2 regulatory subunit b’gamma
PRAD: Prostate cancer
PRKY: Protein kinase Y-linked
PRRX1: Paired related homeobox 1
PSA: Prostate-specific antigen
PSMA: Prostate-specific membrane antigen
PTC: Papillary thyroid carcinoma
PTCL: Peripheral T-cell lymphoma
PTEN: Phosphatase and tensin homolog
PTGER4: Prostaglandin E receptor 4
PTPRO: Protein tyrosine phosphatase receptor type O
PX1: PX Domain containing 1
qDMA-HP: Quantitative DNA melting analysis with hybridization probes
qMS-PCR: Quantitative methylation-specific PCR
qPCR: Quantitative PCR
R: Recruiting
RALYL: RALY heterogeneous nuclear ribonucleoprotein like
RANBP3: RAN binding protein 3
RASSF1: Ras association domain family member 1
RASSF1A: Ras association domain family member 1A
RASSF2: Ras association domain family member 2
RBC IT: Red blood cell transfusion independence
RFS: Recurrence-free survival
RG108: N-phthaloy-L-tryptophan
RIN1: Ras and rab interactor 1
RNF43: Ring finger protein 43
RNF135: Ring finger protein 135
RNF180: Ring finger protein 180
RP: Radical prostatectomy
RRBS: Reduced representation bisulfite sequencing
RT-PCR: Real-time PCR
RUNX1: RUNX family transcription factor 1
RUNX3: RUNX family transcription factor 3
RXFP3: Relaxin/insulin-like family peptide receptor 3
RXR: Retinoid X receptor
SAH: S-adenosylhomocysteine
SAM: S-adenosyl-L-methionine
SARC: Sarcoma
SCNEC: Small cell neuroendocrine carcinoma
SCNN1B: Sodium channel epithelial 1 subunit beta
SCT: Secretin
SDC2: Syndecan 2
SE: Sensitivity
SEPT9: Septin 9
SERPINA1: Serpin family A member 1
SGI-110: Guadecitabine
sgRNA: Single guide RNA
SHOX2: Short stature homeobox 2
SLC5A8: Solute carrier family 5 member 8
SLC7A8: Solute carrier family 7 member 8
SLC31A1: Solute carrier family 31 member 1
SNORD3F: Small nucleolar RNA C/D Box 3F
SOCS3: Suppressor of cytokine signaling 3
SOD3: Superoxide dismutase 3
SORT1: Sortilin 1
SOX17: SRY-Box transcription factor 17
SP: Specificity
SRCIN1: SRC kinase signaling inhibitor 1
SRF: Serum response factor
SST: Somatostatin
STAP1: Signal transducing adaptor family member 1
T: Terminated
TAC1: Tachykinin precursor 1
TALE: Transcription activator-like effector
T-ALL: T-cell acute lymphoblastic leukemia
TBX15: T-box transcription factor 15
TdCyd: 4′-thio-2′-deoxycytidine
TDG: Thymine DNA glycosylase
TERT: Telomerase reverse transcriptase
TET: Ten-eleven translocation
TF: Transcription factor
TFPI2: Tissue factor pathway inhibitor 2
THCA: Thyroid carcinoma
ThinPrep: ThinPrep Pap test
TNFRFS10c: TNF receptor superfamily member 10b
TOP2A: DNA topoisomerase II alpha
TP53: Tumor protein P53
TRDJ3: T-cell receptor delta joining 3
TRHDE: Thyrotropin-releasing hormone degrading enzyme
TRIP13: Thyroid hormone receptor interactor 13
TSHR: Thyroid-stimulating hormone receptor
TSS: Transcription start site
TTS: Transcription termination site
TUSC3: Tumor suppressor candidate 3
TWF2: Twinfilin actin-binding protein 2
TWIST1: Twist-related protein 1
UPF3A: UPF3 regulator of nonsense-mediated MRNA decay homolog A
US: Unknown status
USP44: Ubiquitin-specific peptidase 44
VIM: Vimentin
VTRNA2-1: Vault RNA 2–1
WGBS: Whole-genome bisulfite sequencing
WIF1: WNT inhibitory factor 1
WNT5A: Wnt family member 5A
XCI: X Chromosome inactivation
Xi: Inactive X chromosome
ZEB2: Zinc finger E-box binding homeobox 2
ZFP: Zinc finger protein
ZFP42: Zinc finger protein 42
ZIC1: Zinc finger of the cerebellum 1
ZNF345: Zinc finger protein 345
ZNF382: Zinc finger protein 382
ZNF569: Zinc finger protein 569
ZNF577: Zinc finger protein 577
ZNF582: Zinc finger protein 582
ZNF671: Zinc finger protein 671
ZNF783: Zinc finger protein 783
ZNF808: Zinc finger protein 808
ZSCAN18: Zinc finger and SCAN domain containing 18
